# Genome-Wide Identification and Analyses of Drought/Salt-Responsive Cytochrome *P450* Genes in *Medicago truncatula*

**DOI:** 10.3390/ijms22189957

**Published:** 2021-09-15

**Authors:** Yaying Xia, Junfeng Yang, Lin Ma, Su Yan, Yongzhen Pang

**Affiliations:** 1 Institute of Animal Science, Chinese Academy of Agricultural Sciences, Beijing 100193, China; xiayayin0703@126.com (Y.X.); fyang63@ibcas.ac.cn (J.Y.); malin@caas.cn (L.M.); 17806284739@163.com (S.Y.); 2 Key Laboratory of Plant Resources and Beijing Botanical Garden, Institute of Botany, Chinese Academy of Sciences, Beijing 100093, China; 3 University of Chinese Academy of Sciences, Beijing 100049, China

**Keywords:** cytochrome P450 monooxygenase, *M. truncatula*, expression profile, co-expression analysis, salt stress, drought stress

## Abstract

Cytochrome P450 monooxygenases (P450s) catalyze a great number of biochemical reactions and play vital roles in plant growth, development and secondary metabolism. As yet, the genome-scale investigation on *P450s* is still lacking in the model legume *Medicago truncatula*. In particular, whether and how many *MtP450s* are involved in drought and salt stresses for *Medicago* growth, development and yield remain unclear. In this study, a total of 346 *MtP450* genes were identified and classified into 10 clans containing 48 families. Among them, sixty-one *MtP450* genes pairs are tandem duplication events and 10 *MtP450* genes are segmental duplication events. *MtP450* genes within one family exhibit high conservation and specificity in intron–exon structure. Meanwhile, many *Mt450* genes displayed tissue-specific expression pattern in various tissues. Specifically, the expression pattern of 204 *Mt450* genes under drought/NaCl treatments were analyzed by using the weighted correlation network analysis (WGCNA). Among them, eight genes (*CYP72A59v1*, *CYP74B4*, *CYP71AU56*, *CYP81E9*, *CYP71A31*, *CYP704G6*, *CYP76Y14*, and *CYP78A126*), and six genes (*CYP83D3*, *CYP76F70*, *CYP72A66*, *CYP76E1*, *CYP74C12*, and *CYP94A52*) were found to be hub genes under drought/NaCl treatments, respectively. The expression levels of these selected hub genes could be induced, respectively, by drought/NaCl treatments, as validated by qPCR analyses, and most of these genes are involved in the secondary metabolism and fatty acid pathways. The genome-wide identification and co-expression analyses of *M. truncatula* *P450* superfamily genes established a gene atlas for a deep and systematic investigation of *P450* genes in *M. truncatula*, and the selected drought-/salt-responsive genes could be utilized for further functional characterization and molecular breeding for resistance in legume crops.

## 1. Introduction

Cytochrome P450 monooxygenases (P450s) are one of the largest enzymatic protein families. P450s catalyze NADPH-and/or O_2_-dependent hydroxylation reaction in primary and secondary metabolism in many organisms. P450s are present in most organisms, from bacteria to plants, animals and humans, and account for about 1% of the protein-coding genes in plants [1,2]. A great number of *P450* genes play crucial roles in diverse plant metabolic pathways, including fatty acid pathways, phenylpropanoid pathways, terpenoid pathways, and plant-hormones pathways [3,4]. Meanwhile, P450s are also implicated in many stress responses, such as those to drought, salinity, chemical toxicity, oxidative stress and pest infestation [5].

Structurally, bacterial P450s are soluble proteins, but all P450s of plant origin thus far studied are membrane-localized, and they are usually anchored on the cytoplastic surface of the endoplasmic reticulum by N-terminal transmembrane helix [6]. In some cases, plant P450s with potential signal peptides can be targeted to the plastids or to the mitochondria [7]. A few motifs display high conservation in P450, namely, the proline-rich membrane hinge, the sequence surrounding the cysteine, which is the axial ligand to the heme, the heme-binding motif FXXGXRXCXG (CXG motif), the EXXR motif in the K-helix, the PXRX motif and the AGxD/ET motif associated with oxygen binding and activation in the I-helix. Generally, the E-R-R triad (EXXR and PXRX) is required for locking the heme pockets into position, and assures the stabilization of core structure. Among these conserved domains, only the E-R-R triad and the cysteine in the heme-binding domain are conserved in all plant P450 sequences, and they are signature motif of plant P450s [5,8,9].

Several studies have demonstrated that *P450* genes play significant roles in enhancing plant adaptation to various abiotic stress factors. For an instance, the *Arabidopsis cyp709b3* mutant is sensitive to ABA and salt-stress response, and its over-expression enhanced salt-stress tolerance in transgenic *Arabidopsis* [10]. In another study, the over-expression of *PgCYP736B* gene from *Panax ginseng* conferred enhanced resistance to salt stress in *Arabidopsis* via decreased accumulation of hydrogen peroxide, increased carotenoid levels and the increased expression of the ABA biosynthesis gene [11]. In cotton, when the expression levels of *CYP75A3* and *CYP73A11* genes were suppressed, the hydrogen peroxide level in the silenced plants increased with a significant reduction in glutathione, ascorbate peroxidase and proline under drought and salt-stress conditions. Meanwhile, the knockdown of the *CYP75A3* and *CYP73A11* in cotton also resulted in the down-regulation of several stress-responsive genes, and thus reduced the tolerance of the silenced cotton against salt and drought stress [12].

Legume crops account for one third of the primary crop production in the world, and they have significant impacts on agriculture, the environment and the nutrition of both animals and humans [13]. Drought and salinity stresses are the main abiotic stress factors that have highly negative effects on plant growth and development, and limited the yields of legume crops [12,14]. Among legume plants, *M. truncatula* is an annual species, with the advantages of small diploid genome and easy transformation system [15]. Moreover, *M. truncatula* has been developed as a model plant for the functional genomics of legume plants [16]. Although 151 *P450* genes were identified from EST sequences of *M. truncatula* more than ten years ago [17], information on *P450* genes have been updated and a more comprehensive analysis on *P450* gene is essential for their functional characterization. In this study, we identified and analyzed all *P450* superfamily genes from *M. truncatula*, including gene nomenclature, location, evolution, structure and expression profiles by using various bioinformatics tools. More importantly, we identified drought-/salt-responsive *P450* genes by co-expression analyses, which were also validated by qPCR analyses. Our studies on *CYP450* genes in *M. truncatula* provide basic information for further investigation on their physiological and biochemical functions, as well as for their application in improving drought and/or salt-stress tolerance in legume plants.

## 2. Results

### 2.1. Identification and Analyses of MtP450s Protein Sequences

Previously, a large number of *P450* genes from *M. truncatula* have been deposited at the cytochrome P450 database at the website http://drnelson.uthsc.edu/CytochromeP450.html (accessed on 20 June 2015) following the completion of the *M. truncatula* genome sequencing. In this study, a total of unique 444 *P450* EST sequences were retrieved from the genome database of *M. truncatula*, but 54 of them were pseudogenes and 44 lacked the complete signature motifs of P450, and (98) were thus discarded in the present study. Eventually, a total of 346 *MtP450* genes were selected for further analyses hereinafter (Table S1). Among them, the CYP71 family is the largest, with 59 members, while the families of CYP51, CYP87, CYP701, CYP703, CYP709, CYP710, CYP718, CYP720, CYP727, CYP733 and CYP734 each have only one single gene. The length of the deduced MtP450 protein sequences range from 195 (CYP728B29) to 689 (CYP97A10) amino acids, which correspond to protein molecular weights of 21.69 to 76.98 kDa. The predicted cellular location showed that most MtP450 proteins were located in the plasma membrane (274), some in endoplasmic reticulum (59), a few in membrane bound chloroplast (7), extracellular (4) and cytoplasmic (2) (Table S1).

*P450* genes of *M. truncatula* were also compared with several model plant species, including soybean, *Arabidopsis*, poplar, rice, wheat and maize (Tables S2 and S3, Figure S1). We found that the families of CYP82, CYP83, CYP716, CYP718 and CYP720 only exist in dicotyledons (*M. truncatula*, soybean, *Arabidopsis* and poplar), while CYP99 was shared by rice, wheat and maize. In comparison with the other model legume plant, the soybean, one gene from CYP709 family and three genes from CYP729 family are specific in *M. truncatula*.

### 2.2. Classification and Phylogenetic Relationship of MtP450 Proteins

**Figure 1:**
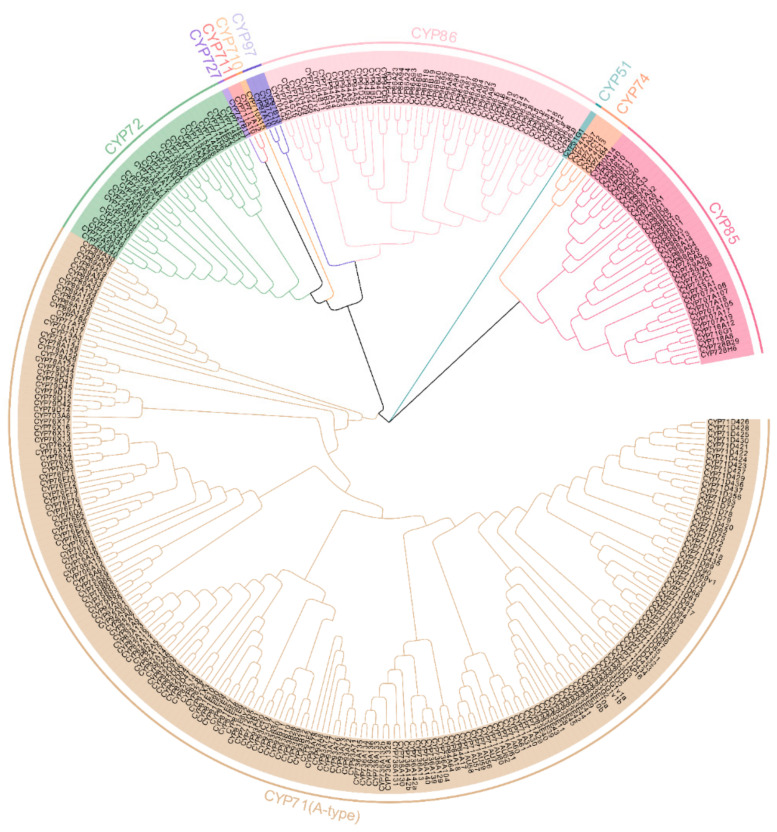
Phylogenetic relationships of MtP450 proteins. An unrooted NJ tree of MtP450s was constructed using the MEGA-X software, bootstrap used was 1000 replicates. The entire MtP450 family members are shown, for each clan, with different colors.

All the 346 *MtP450* genes were assigned to 48 families according to the P450 nomenclature. They can be classified into A-type (19 families, Table S2) with 209 genes, and non-A type (29 families, Table S3) with 137 genes. To explore the evolutionary relationships among MtP450s, an unrooted NJ tree with the 346 full-length MtP450s sequences was constructed (Figure 1). Based on the phylogenetic analysis, MtP450s were classified into 10 clans, including 6 single-family clans (CYP711, CYP727, CYP710, CYP97, CYP51, and CYP74) and 4 multi-family clans (CYP71, CYP72, CYP86, and CYP85, in descending order, by number). Alternatively, these ten clans could be grouped into three clusters: CYP51, CYP74, and CYP85 clans formed one cluster, of which the CYP51 clan is likely to be the oldest; CYP72, CYP727, CYP711, CYP710, CYP97 and CYP86 clans were tightly clustered together to form another cluster, and CYP71 clan is considered to be a special cluster with the most members (Figure 1).

### 2.3. Location and Syntenic Analyses of MtP450 Genes

**Figure 2:**
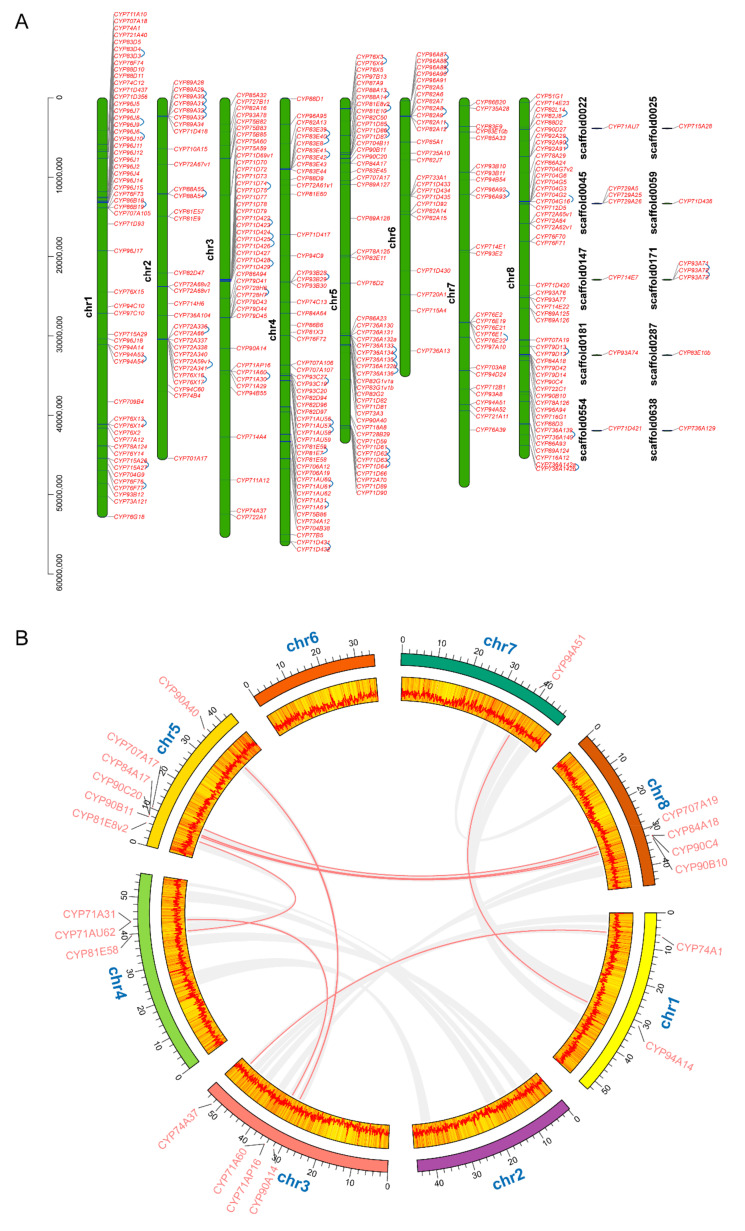
Chromosomal localization and gene duplication analysis of *MtP450* genes. (**A**) All 346 *MtP450s* are shown on the chromosomes and indicated by their names. Chromosome numbers are indicated on the left side of each bar. Tandem duplicated genes are joined with blue arc lines. (**B**) The gray lines in the background indicate the collinear blocks within *M. truncatula*, while the red lines highlight the segmental duplication of *MtP450* gene pairs. The yellow background on the chromosomes represents the gene density at each chromosomal position, and the vertical red line inside represents relatively higher gene density. The horizontal red line also represents gene density at each chromosomal position, with peak values representing relatively higher gene density and valley values representing relatively lower gene density.

To further investigate the evolutionary mechanism of *MtP450* genes family, both tandem and segmental duplication events were analyzed. In total, three hundred thirty-two *MtP450* genes distribute on 8 chromosomes, and 14 genes on 10 scaffolds. A relatively high gene density was observed in chromosomes Nos. 1, 3, 4, 5, and 8 (Figure 2A). Sixty-one *MtP450* genes pairs can be identified as tandem duplication (Figure 2A and Table S4), and 10 genes as segmental duplication (Figure 2B and Table S4). These results indicated that tandem duplication was the major driving force for the augmentation of the *MtP450s* superfamily. 

**Figure 3:**
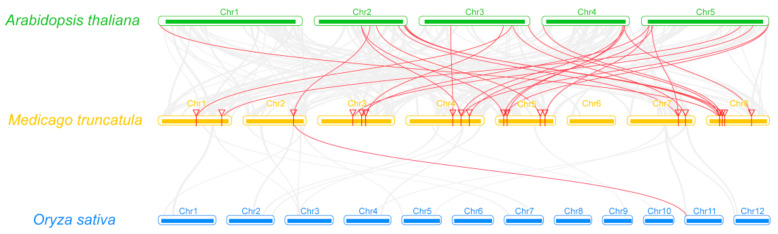
Synteny analysis of *MtP450* genes between *M. truncatula* and two representative plant species (*A. thaliana* and *O. sativa*). Gray lines in the background indicate the collinear blocks within *M. truncatula*, and *A. thaliana*/*O. sativa*, while the red lines highlight the syntenic *MtP450* gene pairs.

Furthermore, three comparative syntenic maps of *M. truncatula*, with two representative plant species *Arabidopsis* for dicot and rice for monocot, were constructed to illustrate the evolution mechanism of *MtP450s* family genes. Thirty-six orthologous pairs were found between *Medicago* and *Arabidopsis*, but only one orthologous pair were found between *Medicago* and rice (Figure 3, Tables S5 and S6).

In order to better understand the effect of evolutionary stress on the formation of *MtP450* gene family, Ka/Ks analysis on the *MtP450* gene pairs were analyzed. Most *MtP450s* are tandem and segmental duplicated gene pairs (Table S4), and all the orthologous *MtP450* gene pairs had Ka/Ks value of less than 1 (Tables S5 and S6). These results clearly indicated that *MtP450* genes might have undergone strong purifying selective pressure during evolution, except CYP83E39 and CYP83E40 with Ka/Ks value of 2.020 that might have undergone positive selection.

### 2.4. Analyses of the Structure of MtP450 Genes

**Figure 4:**
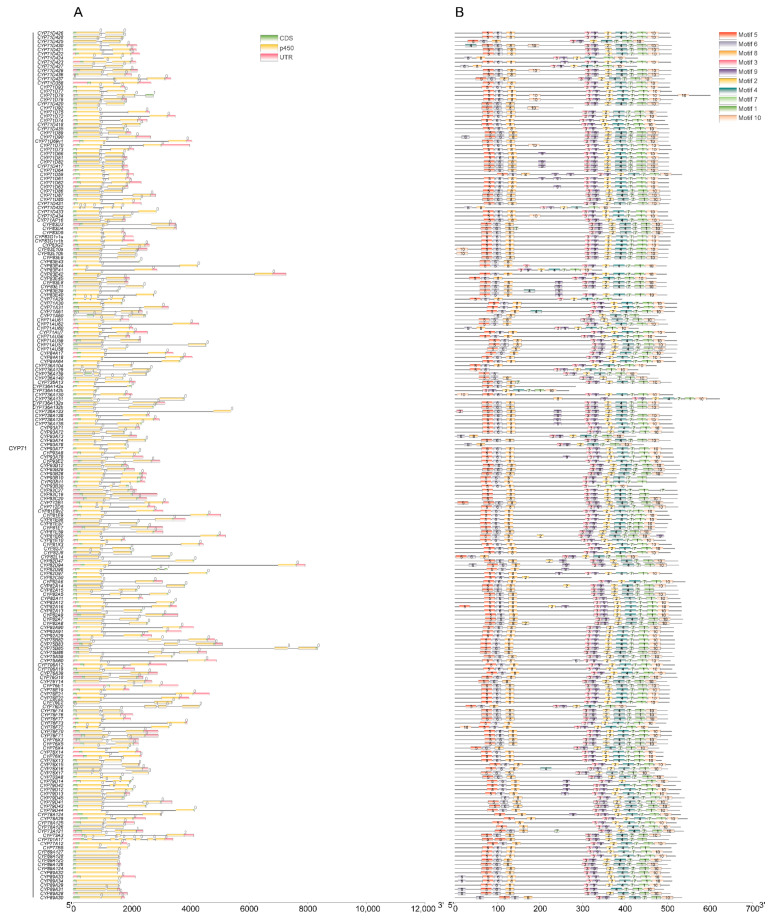

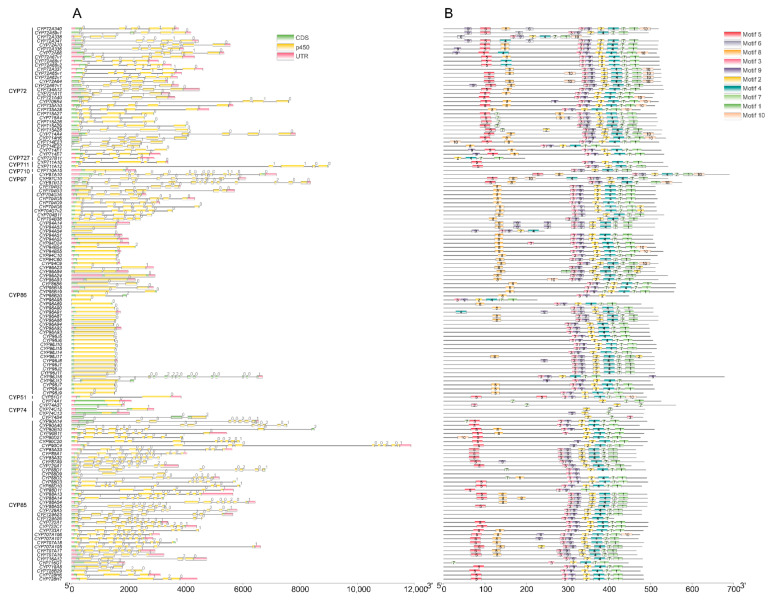
Gene structural features and conserved motifs of MtP450 proteins. (**A**) Gene structural features of *MtP450s*. “p450” indicate the conserved domain of Cytochrome p450 family, “CDS” indicated the remaining partial coding sequence. “UTR” indicates untranslated regions. The numbers 0, 1, and 2 stand for the types of intron phase. The length of the intron, exon and UTR can be estimated from the scale. (**B**) Analysis of the conserved motifs of MtP450 proteins. Each motif is represented by a colored box. Box length corresponds to motif length.

To understand the structural features of *MtP450* genes, intron–exon structures were detected based on their evolutionary relationships. Some families contain intronless genes, such as all members of the CYP710 clan and most members of the CYP89, CYP94 and CYP96 clans (except *CYP89A29*, *CYP89A31*, *CYP94B54*, *CYP94C9*, *CYP96J18*, *CYP96J12*, and *CYP96J9*) (Figure 4A). The majority of CYP71 clan genes (194 out of 209), have only a single phase-zero intron, implying their close phylogenetic relationship (Figure 4A). Intron positions and phases are conserved within most members of a single family, such as clans of CYP81, CYP83, CYP84, CYP92, CYP96 and CYP706. Some members have more than ten introns, for examples, *CYP97B13*, *CYP96J18*, and *CYP97A10* are interrupted by 13, 13 and 14 introns, respectively (Figure 4A). Most members of the CYP85 clan have up to seven or eight introns, some have two or three introns (eg. CYP716, CYP718 and CYP728 clans). In particular, the CYP72 clan is characterized by three or four introns with relatively stable position and phase, except *CYP72A338* with two introns and different phases (Figure 4A). 

Analysis on conserved motifs is one of the important methods for sequence-based protein-function prediction [5]. To identify conservative structures of MtP450 proteins, key motifs were searched via MEME motif analysis, and 10 motifs were found for *MtP450* genes (Figure 4B and Table S7). It showed that the majority of the CYP71-clan proteins contained all these 10 motifs, while only 37 of them have less than 10 motifs. The CYP72-clan proteins contained 7–9 motifs, except for CYP72A340 with 10 motifs. The CYP85 clan contained 6–7 motifs, apart from CYP88D9 (2), CYP88D11 (4), CYP729A26 (5) and CYP716G1 (5). The CYP86 clan contained 6–8 motifs, except CYP94A54 (4), CYP86B20 (4), and CYP96J12 (5). Most CYP74-clan members contained 2–3 motifs, but CYP51, CYP97, CYP710, CYP711, CYP727 genes had 8, 9, 6, 7, 4 motifs, respectively (Figure 4B). Most MtP450 proteins within the same clan possess same motifs, indicating that these MtP450s members may function similarly.

### 2.5. Analysis of the Cis-Regulatory Elements of MtP450 Genes

The *cis* elements in promoters are important for transcriptional regulation and gene function. A total of 15 putative *cis* elements were found in 346 *MtP450s* by using the PlantCARE online database. Briefly, ten hormone-responsive elements, including auxin responsiveness (TGA-element, AuxRR-core), MeJA responsiveness (CGTCA-motif, TGACG-motif), GA responsiveness (GARE, P-box, TATC-box), SA responsiveness (TCA-element), ethylene responsiveness (ERE) and ABA/JA responsiveness (ABRE) were identified in their promoter regions (Figure S2). In addition, five abiotic and biotic stress-responsive regulatory elements involved in drought responsiveness (MBS), wound responsiveness (WUN motif), stress responsiveness (TC-rich repeats), low-temperature responsiveness (LTR) and anaerobic induction (ARE) were also detected for individual *MtP450* genes (Figure S2). However, the number and position of these *cis* elements varied for different *MtP450* genes. The presence of a large number of phytohormone-responsive elements and stress-responsive regulatory elements in the promoters of *MtP450* genes suggested that they play important and diverse roles in growth and development processes in *M. truncatula*.

### 2.6. Expression Profiles of MtP450 Genes

**Figure 5:**
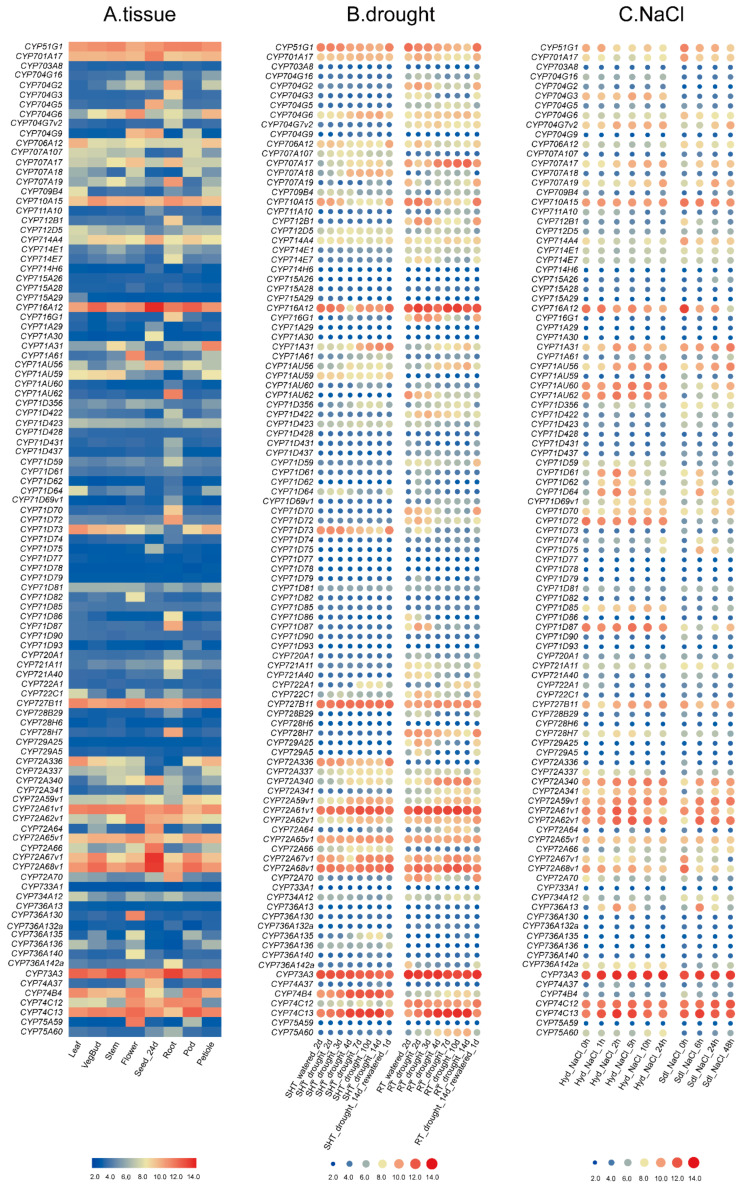

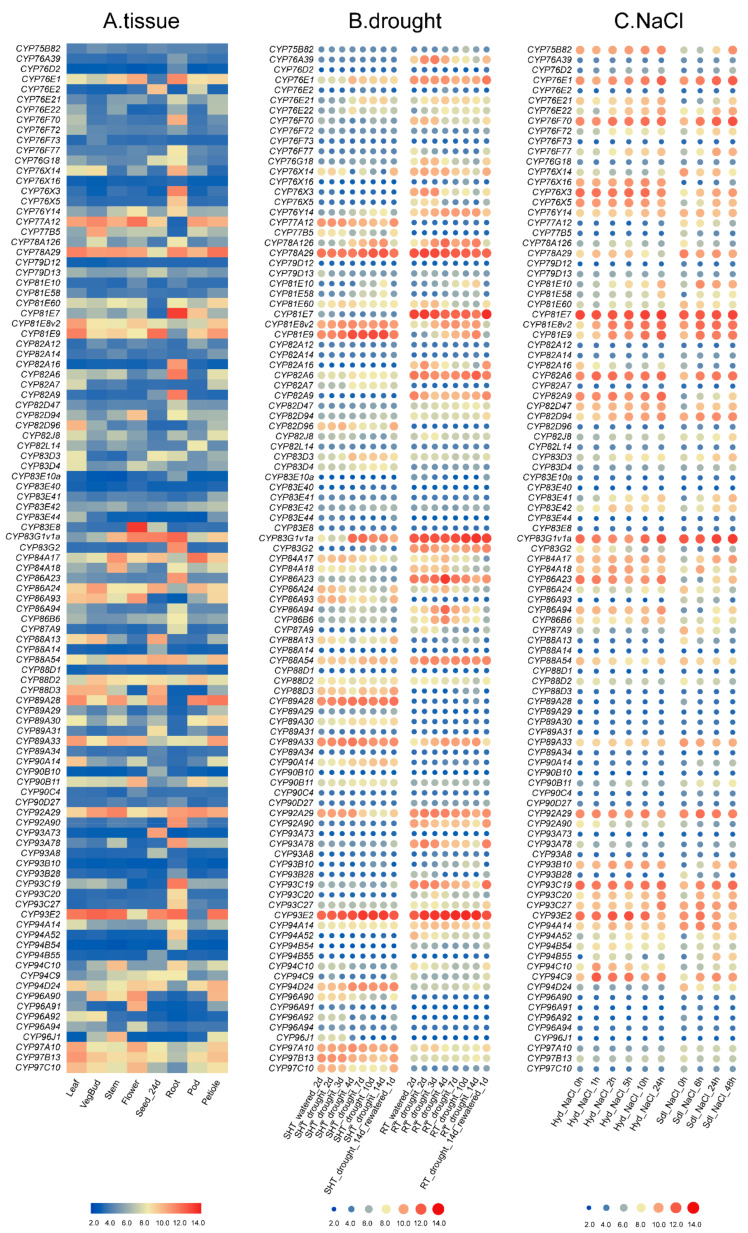
Expression profiles of *MtP450* genes. (**A**) Expression profiles of *MtP450* genes in eight different tissues from microarray data. (**B**,**C**) are hierarchical clustering of gene expression profiles under drought (**B**) and NaCl (**C**) treatments from microarray data. Each column indicates a sampling time point, and each row indicates an *MtP450* gene, wherein each treatment was normalized in the same row, the relative expressions levels are log2-transformed and visualized by heatmap. The colors vary from blue to red, and circles from small to large represent the scale of relative expression levels. Those genes with deeper background color showed that their expression was significantly increased after stress induction. “SHT” and “RT” stand for “shoot” and “root” materials, respectively, used for drought treatment; “Hyd” and “Sdl” stand for hydroponic treatment with 200 mM NaCl, and in vitro culture with 180 mM NaCl, respectively.

To gain more insight into the roles of *MtP450s* in *M. truncatula* growth and development, the expression patterns of *MtP450s* were analyzed in major tissues, including roots, stems, leaves, petioles, vegetative buds, flowers, seed pods and seeds at 24 dap (days after pollination) for 204 *MtP450* genes (out of all 346 *MtP450* genes) with available microarray data (Figure 5A, additional file 1).

Among them, thirteen *MtP450* genes (*CYP51G1*, *CYP701A17*, *CYP710A15*, *CYP716A12*, *CYP727B11*, *CYP72A61V1*, *CYP72A65V1*, *CYP72A67V1*, *CYP72A68V1*, *CYP73A3*, *CYP74C13*, *CYP78A29*, and *CYP92A29*) were highly expressed in all eight tested tissues (Figure 5A). Seventeen genes (*CYP94B54*, *CYP716G1*, *CYP76X5*, *CYP71D704G3*, *CYP93C20*, *CYP71D86*, *CYP94A52*, *CYP82A9*, *CYP83G2*, *CYP82A16*, *CYP71AU62*, *CYP76X3*, *CYP712B1*, *CYP93C27*, *CYP92A90*, *CYP71D87*, and *CYP728H7*) were highly expressed in roots, five (*CYP83E8*, *CYP75A59*, *CYP71A61*, *CYP736A130*, and *CYP96A91*) in flowers, three (*CYP71A30*, *CYP93A73*, and *CYP89A31*) in 24-dap seeds (Figure 5A). In addition, *CYP77A12*, *CYP81E9* and *CYP89A28* were highly expressed in seven other tissues except roots, and *CYP93E2* was highly expressed in seven other tissues except seed pods (Figure 5A).

Drought is one of the major abiotic stresses resulting in yield losses and restricted plant cultivation. To characterize possible roles of *MtP450s* in response to drought stress, the transcript abundance of these 204 *MtP450* genes were also analyzed under drought stresses in shoots and roots retrieved from available microarray data. Overall, these genes exhibited distinct expression profiles under drought stress, either in shoots or in roots (Figure 5B). Notably, at least eight genes were highly expressed in both shoots and roots (eg. *CYP51G1*, *CYP716A12*, *CYP72A61V1*, *CYP72A68V1*, *CYB73A3*, *CYP74C13*, *CYP78A29* and *CYP93E2*). In particular, the expression of eight *MtP450s* (*CYP704G6*, *CYP707A17*, *CYP707A18*, *CYP72A340*, *CYP72A59V1*, *CYP76E21*, *CYP78A126*, and *CYP81E9*) shared the same pattern, gradually increased during drought treatment, but declined once re-watered in both shoots and roots (Figure 5B). In addition, the expression of six genes (*CYP72A62V1*, *CYP76E1*, *CYP76E21*, *CYP83D3*, *CYP83D4* and *CYP83G1V1a*) in shoots, and other three genes (*CYP704G7V2*, *CYP72A64* and *CYP81E8V2*) in roots also shared the same pattern (Figure 5B), and these genes may play important roles in responses to drought stress.

Salt stress great impairs plant production and quality by limiting their growth and development. The transcript abundance of all *MtP450* genes (the same set of microarray probes) were also analyzed under salt stress, in roots treated with 180 mM NaCl in vitro culture or with 200 mM NaCl in hydroponics culture (Figure 5C). It was clear that each gene responded differently to salt treatment (Figure 5C). Specifically, the expression levels of at least 24 genes (*CYP704G7V2*, *CYP707A17*, *CYP707A19*, *CYP71AU56*, *CYP71D69V1*, *CYP71D70*, *CYP71D85*, *CYP72A340*, *CYP72A341*, *CYP72A59V1, CYP72A62V1*, *CYP75B82*, *CYP76E21*, *CYP76E22*, *CYP76F72*, *CYP76F77*, *CYP81E10*, *CYP81E58*, *CYP81E8V2*, *CYP81E9*, *CYP82D47*, *CYP82D94*, *CYP83E41* and *CYP93C20*) under both in vitro and hydroponics culture, two genes (*CYP76Y14* and *CYP93C27*) under hydroponics culture, and seven genes (*CYP71AU60*, *CYP71AU62*, *CYP76F70*, *CYP76X3*, *CYP76X5*, *CYP82A6* and *CYP84A17*) in in vitro culture were up-regulated at various level (Figure 5C). These results suggested that the above-mentioned genes are associated with salt stresses in *M. truncatula*.

### 2.7. Co-Expression Analysis of MtP450 Genes under NaCl and Drought Treatments

Weighted gene co-expression network analysis (WGCNA) is a systematic biological method designed to analyze a gene network rather than individual genes [18,19]. To identify potential functional modules participated in drought and/or salt stresses in *M. truncatula*, a total of 204 *MtP450* genes under drought and salt treatment were also subjected to WGCNA.

As observed in the dendrogram of genes under drought treatment, two distinct modules (labeled in different color) were identified in shoots (Figure S3), and three modules in roots (Figure S4). Due to the distinct expression profile of different module’s eigengenes, each module was related to a specific expression-pattern response to drought stress (Figures S3B and S4B). Notably, the gene-expression levels of shoots in the turquoise module gradually increased with treatment time and declined once rewatered (Figure S3B), and genes expressed in roots within the brown module shared a similar expression pattern during drought treatment (Figure S4B). These results suggest that the genes in these modules are most likely involved in drought tolerance in *M. truncatula*. Under NaCl treatment, three unique module eigengenes were obtained (Figure S5A), and these genes, in the brown module, were up-regulated with treatment time (Figure S5B), thus these could possibly participate in NaCl tolerance in *M. truncatula*.

**Figure 6:**
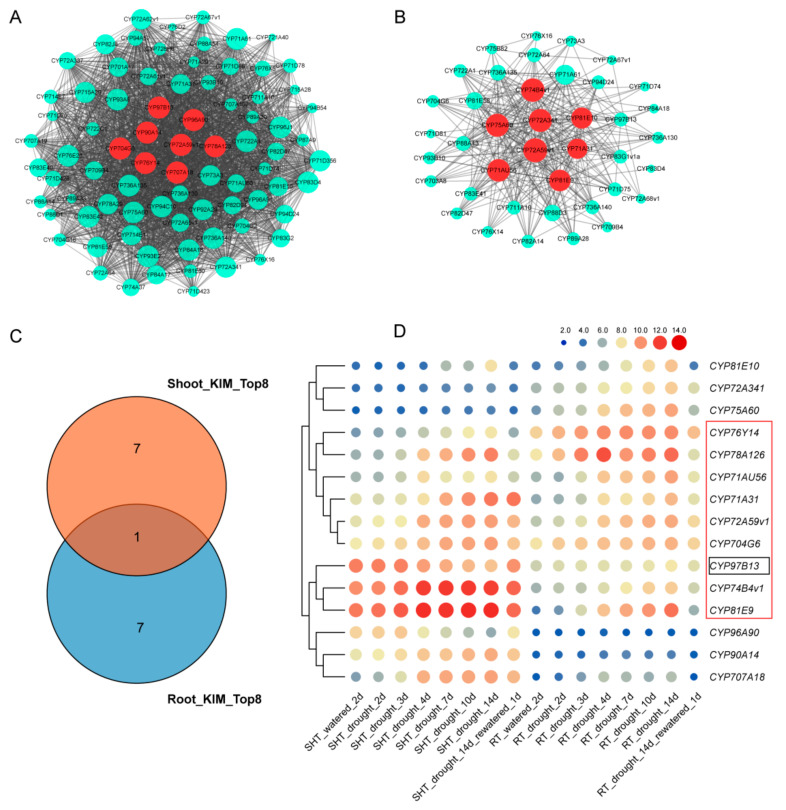
Co-expression network analysis and hierarchical clustering of expression profiles of *MtP450* genes under drought treatments from microarray data. (**A**) Weighted gene co-expression network analysis of genes in shoots; (**B**) weighted gene co-expression network analysis of genes in roots; (**C**) Venn diagram showing *MtP450* genes in shoots and roots under drought treatments through co-expression; (**D**) hierarchical clustering of expression profiles of *MtP450* genes under drought treatments from microarray data.

**Figure 7:**
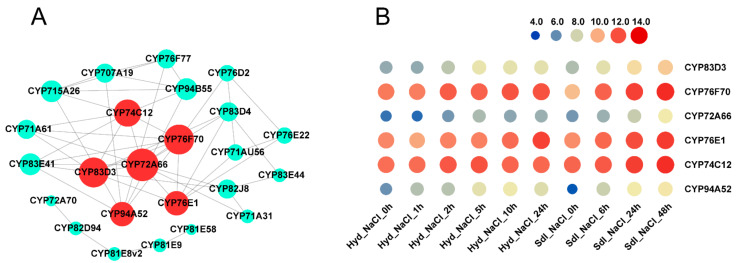
Co-expression network analysis and hierarchical clustering of expression profiles of *MtP450* genes under NaCl stresses. Co-expression network analysis (**A**) and hierarchical clustering of expression profiles (**B**) of *MtP450* genes under NaCl treatments from microarray data.

Furthermore, co-expression networks were constructed to identify candidate *MtP450* genes under drought and salt treatments. In these networks, each node represents a gene and the connecting lines (edges) between genes represent co-expression correlations. Hub genes are those that show most connections in the network. In the drought-responsive module networks (turquoise module in shoots and brown module in roots, Figure S3B and S4B), eight genes in shoots (*CYP97B13*, *CYP96A90*, *CYP90A14*, *CYP704G6*, *CYP72A59v1*, *CYP78A126*, *CYP76Y14* and *CYP707A18*) and another eight genes in roots (*CYP74B4v1*, *CYP75A60*, *CYP72A341*, *CYP81E10*, *CYP72A59v1*, *CYP71A31*, *CYP71AU56* and *CYP81E9*) were predicted to be core hub genes, respectively (Figure 6A,B). In particular, *CYP72A59v1* was the only hub gene from both shoots and roots (Figure 6C). Taken together, our results indicated that the expression levels of eight genes (*CYP72A59v1*, *CYP74B4v1*, *CYP71AU56*, *CYP81E9*, *CYP71A31*, *CYP704G6*, *CYP76Y14* and *CYP78A126*) were highly expressed in shoots and roots under drought treatment, and were decreased once re-watered (Figure 6D), indicating that they may play an important role in responses to drought stress. Similarly, six hub genes (*CYP83D3*, *CYP76F70*, *CYP72A66*, *CYP76E1*, *CYP74C12* and *CYP94A52*) were obtained in the salt-responsive module networks (brown module in Figure S5A and Figure 7A), which showed high expression level after NaCl induction (Figure 7B). Therefore, eight hub genes associated with drought stress and six hub genes associated with salt stress were further selected for validation by qPCR.

### 2.8. Analysis of Hub Genes under Drought and Salt Treatment by qPCR

In order to experimentally validate the involvement of the above-mentioned fourteen genes induced under drought/salt stresses, their expression levels at 0 h, 3 h, 24 h, 48 h and 72 h were analyzed via qPCR.

**Figure 8:**
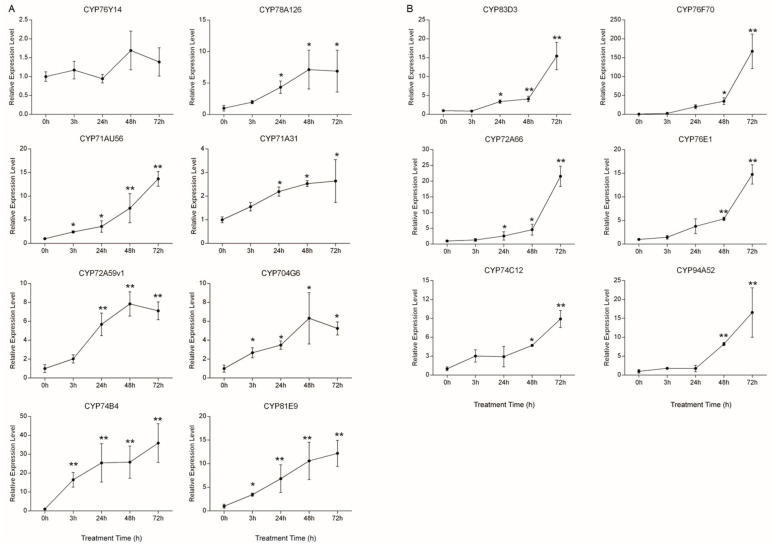
Analysis of the 14 hub genes under drought/salt treatments by qPCR. (**A**) Relative expression levels of eight hub *MtP450* genes under drought treatment at 3h, 24h, 48 h and 72 h, with that of 0h as control (set as value of 1). (**B**) Relative expression levels of six hub *MtP450* genes under salt treatment at 3 h, 24 h, 48 h and 72 h, with that of 0h as control (set as value of 1). The differential expression analysis was conducted based on the 2^−∆∆ct^ method. Data are from three biological replicates and three technical replicates. * *p* < 0.05, ** *p* < 0.01.

Under drought treatment (treated with PEG), the expression levels of *CYP71AU56*, *CYP71A31*, *CYP74B4* and *CYP81E9* genes gradually increased from 3 h, 24 h and 48 h to 72 h, but those of *CYP704G6*, *CYP72A59v1* and *CYP78A126* only increased at 3 h, 24 h and 48 h, declining at 72 h, while that of *CYP76Y14* fluctuated at different time points and its expression level was highest at 48 h (Figure 8A).

For the NaCl treatment, the expression levels of *CYP83D3, CYP76F70*, *CYP72A66* and *CYP76E1* increased slowly from 3 h and 24 h to 48 h, but increased sharply from 48 h to 72 h when compared with the control at 0 h (Figure 8B). Meanwhile, the expression level of both *CYP74C12* and *CYP94A52* increased more sharply at 24 h than the other four genes (Figure 8B). Our results demonstrated that these fourteen genes were all responsive to drought/salt stress, which was consistent with the co-expression analyses.

## 3. Discussion

### 3.1. Comprehensive Identification of P450 Genes in M. truncatula

In the present study, a comprehensive database of gene nomenclature, evolutionary relationships and expression profiles of *P450* genes was established in *M. truncatula*. In total, we identified 346 *P450* genes in the *M. truncatula* genome, and all the *P450* genes were individually designated with unique names by the nomenclature system. The *P450* genes represent approximately 0.6% of the *Medicago* genome, which is between the average of 0.1–1% for the *P450* gene in other plant species [2]. All the *MtP450* genes were classified into 10 clans and 48 families. In contrast, less than half (151) putative *P450s* genes were identified for *M. truncatula* in the previous study [17], and they were clustered into only 9 clans and 44 families. Clan CYP727, and the subfamilies CYP75, CYP718, CYP728 and CYP733 are the novel clan and subfamilies identified in the present study. The reason for the identification of fewer *P450* genes in the previous study could be explained by the only tentative consensus sequences used, in contrast to the whole-genome sequence used here. In addition, the number of the *P450* genes is also larger in *M. truncatula* than in *Arabidopsis*, with 264 genes and 47 families, which might be due to gene expansion in *M. truncatula* for the production of diverse compounds. The number of *P450* genes in *M. truncatula* were compared to several model plant species, including soybean, *Arabidopsis*, rice, poplar, wheat and maize (Tables S2 and S3, Figure S1). We found that *M. truncatula* had the most P450 families—except for CYP724 and CYP98—as shared with other plant species. In comparison with soybean, one gene from the CYP72 family and three genes from CYP729 family seemed to be unique in *M. truncatula*, which is absent in soybean, as found in previous analyses [20].

P450s from plants are generally classified into two main clades: the non-A type and the A-type. The non-A type contains 10 clans (CYP51, CYP72, CYP74, CYP85, CYP86, CYP97, CYP710, CYP711, CYP727 and CYP746), whereas the A-type only contains one clan, CYP71 [21]. Out of the 10 clans of non-A-type P450s, CYP746 was absent in all seven plant species, including *M. truncatula*, soybean, rice, *Arabidopsis*, poplar, wheat and maize (Table S2 and S3, Figure S1). Non-A type P450s have more diverse compositions than A-type, which may be evolutionally more ancient than A-type, allowing more time for gene duplication and rearrangement [20]. In *M. truncatula*, the A-type *P450* genes accounts for more than 60% (209 out of 346), which is similar to that of *Arabidopsis* (63%, 167 out of 264), indicating that *M. truncatula* has evolved a great number of P450s for the biosynthesis of diverse metabolites, just as has *Arabidopsis* [8].

### 3.2. Non-A-Type P450 Involved in Drought and/or NaCl Stresses

Soil salinity and drought are global problems limiting land usage and crop production. It was estimated that approximately one-fifth of the irrigated lands in the world is affected by salinity [22]. In this study, we found fourteen *MtP450* hub genes responded to drought/NaCl stresses through WGCNA. Among them, six hub genes are non-A type, namely, *CYP72A59v1*, *CYB74B4*, and *CYP704G6* were responsive to drought treatment, and *CYP72A66*, *CYP74C12* and *CYP94A52* to salt treatment (Figure 8). *CYP72A59v1* and *CYP72A66* were from the CYP72 clan, *CYB74B4* and *CYP74C12* from the CYP74 clan, and *CYP704G6* and *CYP94A52* from the CYP86 clan, respectively (Figure 1).

CYP72 clan is associated with the biosynthesis of fatty acids, terpenoids and hormones (BRs, GAs and CKs) [23,24]. Most genes of the CYP72A subfamily were shown to be clustered in tandem arrays on chromosomes 2 and 8 in *M. truncatula*, four CYP72As (*CYP72A61*, *CYP72A63*, *CYP72A67* and *CYP72A68*) were reported to be involved in oleanane triterpene scaffold oxidation [25,26,27]. CYP72A61, a C22-hydroxylase of the non-hemolytic saponin biosynthesis pathway, converted 24-hydroxy-β-amyrin to soyasapogenol B. CYP72A67 and CYP72A68 were found to be C2β-hydroxylase and C23-oxidase of the hemolytic saponin pathway, respectively [27,28]. CYP72A63 was found to possess C30-oxidase activity and it can convert β-amyrin to 11-deoxoglycyrrhetinic acid of the triterpene saponins [26]. Glycyrrhizin of triterpenoid saponin and its hydrolysed metabolite, 18β-glycyrrhetinic acid from *G. glabra*, play an important role in ROS scavenging to reduce oxidative damage [29]. In vitro drought stress via PEG treatment increased saponin content and antioxidant activity in *Agave salmiana* plants [30]. In the genus *Medicago*, saponins are a complex mixture of oleanane-type triterpenic glycosides were both constitutively synthesized and induced in response to biotic/abiotic stresses [31]. In the present study, the expression level of *CYP72A59v1* was increased under drought stress and extremely significant increased under PEG treated at 24 h,48 h and 72h, and the expression level of *CYP72A66* was significantly increased from 24 h under NaCl treatment, suggesting that both *CYP72A59v1* and *CYP72A66* are involved in saponin biosynthesis in response to drought/salt stress in *M. truncatula*.

CYP74s clans are involved in the formation of plant oxylipins, a collective term of oxygenated metabolites derived from polyunsaturated fatty acids (PUFAs) [32]. In *Arabidopsis*, *CYP74A1* encodes an allene oxide synthase (AOS), which functions in transforming 13-hydroperoxy linolenic acid (13-HPOT) to plant defense hormone, jasmonic acid [33]. CYP74B2 is a hydroperoxide lyase (HPL) in *Arabidopsis*, which converts 13HPOT to 6-carbon aldehydes and 12-carbon ω-keto fatty acids [34]. CYP74B4 (*MtHPL3*) encodes a functional 13-HPL, which is similar to 13-HPL from other plant species [35]. CYP74C12 (MtHPL2) and CYP74C13 (MtHPL1) ascribed to the 9/13-HPL subfamily [36,37]. Most MtHPL act as important regulators of plant defense response to (a)biotic stresses. In addition, *CYP74C12* (*MtHPL2*) together with *MtHPL1* and *MtHPL4* locally reacted to salt and wounding stress in roots [37], which is consistent with our results that *CYP74C12* is responsive to salt stress (Figure 8).

CYP94s were also involved in the oxylipin biosynthetic pathway. For example, CYP94A1 catalyzed fatty acid (FA) co-hydroxylation in *Vicia sativa* and CYP94A5 catalyzed the v-hydroxylation of saturated and unsaturated fatty acids in *Nicotiana tabacum* [38,39]. In *Arabidopsis*, CYP94B3 and CYP94C1 oxidize JA-Ile at the C12 position, the over-expression of both CYP94B3 and CYP94C1 led to a reduced response to JA [40]. Increased expression levels of *CYP94C2b* promoted JA inactivation and partially suppressed expression of JA-responsive genes induced by salt treatment. In addition, transgenic rice lines over-expressing *CYP94C2b* showed delayed leaf senescence and enhanced shoot viability under salinity stress condition [41]. In our study, *CYP94A52* showed a high expression level in roots with a TC-rich repeats in the promoter region, which may be involved in response to salt stress.

*CYP704G6* is the orthologous gene of *AtCYP704A2*, and CYP704B1 in the same clan could hydroxylate saturated, unsaturated and epoxy C16 and C18 fatty acids in the ω position [18]. In rice, CYP704B2 functions as CYP704B1, and the deletion of CYP704B2 disturbs the pollen exine and anther cuticle development by blocking the ω-hydroxylation of fatty acids in rice [42]. In *M. truncatula*, *CYP704G6* showed high expression level in flowers, which is likely involved in fatty acid biosynthesis in flower and in response to drought stress during flower development as CYP704B2.

### 3.3. A-Type P450 Involved in Drought and/or NaCl Stresses

In addition to six non-A-type *MtP450* genes, another eight A-type *MtP450* genes from 71 clan were also found to be the hub genes during drought/salt stresses. *CYP71A31*, *CYP71AU56*, *CYP76Y14* CYP78A126, and *CYP81E9* were responsive to drought stress, and *CYP76F70*, *CYP76E1*, and *CYP83D3* to salt stress (Figure 8).

In *Arabidopsis*, CYP71A12 and CYP71A13 were able to convert indole-3-acetaldoxime (IAOx) to indole-3-acetonitrile (IAN) and further to a-hydroxy-IAN and/or dehydro-IAN in vitro at different efficiencies [43,44]. In addition, CYP71A12 play a role in the pathogen-triggered biosynthesis of indole-3-carboxylic acid (ICA) derivatives (ICAs) [44]. CYP71A27 was a component of the camalexin biosynthesis pathway specifically presented in the roots [45]. In Brassicaceae, IAOx is an intermediate in the biosynthesis of IAA, indole glucosinolates, ICAs, ICNs and camalexin, triggering the relevant metabolic pathway to optimize fitness under changing environment [44,46]. IAN can be converted into IAA by nitrilases (NITs) [47]. Exogenous IAA improved drought tolerance of white clover by affecting plant hormones homeostasis and by regulating genes involving in drought stress response and leaf senescence [48].

*CYP71AU60* and *CYP71AU61* of the CYP71A clan were up-regulated in *M. truncatula* seedlings grown in different N sources (NO^3−^, NH^4+^ and urea) treated with IAOx, and they are probably IAOx dehydratase and responsible for the conversion of IAOx to IAN in IAA biosynthesis [49]. Transcriptome analyses of sorghum plants revealed the up-regulation of *CYP71A25* and *CYP71B2* genes under drought stress, along with other drought-responsive genes [50]. In the present study, the expression levels of *CYP71A31* and *CYP71AU56* were increased under PEG treatment, as revealed by both qPCR and microarray analyses, therefore, CYP71A31 and CYP71AU56 are likely IAOx dehydratase in IAA biosynthesis in response to drought stress in *M. truncatula*.

CYP76s catalyze geraniol hydroxylation and take part in terpenoids biosynthesis in different plant species. For example, CYP76B6 from *C. roseus* catalyzes geraniol 8-hydroxylation and is involved in the biosynthesis of secoiridoids and monoterpene indole alkaloid [51]. The same activity was found in its close relative CYP76B10, from *Swertia mussotii* [52], and CYP76C1, from *Arabidopsis* [53]. In another case, CYP76Fs from sandalwood (*Santalum album*) hydroxylate the sesquiterpenes santalene and bergamotene [54]. Salt stress increased the biosynthesis of terpenoids in both leaves and roots from *K. candel*, and in roots from *B. gymnorrhiza*. Moreover, the proportion of terpenoida was increased significantly with the increase of salt concentration in the roots of *K. candel* and *B. gymnorrhiza*. In addition, drought induced an increase in the sesquiterpene production in *Salvia dolomitica* Codd [55]. Triterpenes and related compounds displayed antioxidant activity to scavenge ROS [56], and they could also protect the photosynthetic apparatus from oxidative stress by quenching reducing power to alleviate drought stress [57,58,59]. In our study, it was found that *CY76Y14*, *CY76F70* and *CY76E1* were all preferentially expressed in roots, which is consistent with the high accumulation of terpenoid compounds in roots of *M. truncatula* [31]. The expression levels of *CYP76F70* and *CYP76E1* were increased after NaCl treatment, and *CYP76Y14* after PEG treatment, therefore, these *MtP450* genes may be involved in terpenoids biosynthesis and respond to salt/drought stress.

It was reported that glucosinolates were accumulated under salinity stress and the increase in glucosinolates was related to the synthesis of osmoprotective compounds. The hydrolysis products of the glucosinolates (their cognate isothiocyanates) could lead to the inhibition of inward K^+^ channels in the guard cells to avoid water loss by stomatal closure [60]. In plants, CYP83B1 is involved in indole glucosinolate biosynthesis and CYP83A1 in aliphatic glucosinolate biosynthesis [61,62]. In our study, the expression level of *CYP83D3* was significantly increased at 48 h and 72 h under NaCl treatment, which may be involved in glucosinolate biosynthesis and in response to salt stress.

*CYP81E9* encodes an isoflavone 3′-hydroxylases (MtI3′H) that showed activity toward the isoflavonoid compounds 2′-hydroxyformononetin, pseudobaptigenin and daidzein [63]. Polyphenols and flavonoids are among the most adaptable natural compounds, enabling plants to scavenge reactive oxygen species (ROS) [64] that were commonly produced under drought stress [65]. CYP81E9 catalyzes the biosynthesis of isoflavone and may enhance the scavenging ROS capability to protect plant against drought stress. In addition, CYP78A126 of *M. truncatula* showed 47.47% sequence similarity with CYP78A9, a flavonoid 3′-monooxygenase in *Arabidopsis* [66], therefore, CYP78A126 is also likely involved in flavonoid pathways and protect plants by reducing the level of ROS under drought stress.

In summary, 14 *MtP450* hub genes are involved in different metabolic pathways, and they function in various processes in response to drought/NaCl stresses, including scavenging ROS, promoting stomatal closure, affecting plant development and hormones homeostasis. Namely, *CYP72A59v1* and *CYP72A66* in saponin pathways, *CYP76F70*, *CYP76E1* and *CYP76Y14* in terpenoid pathways, *CYP81E9* and *CYP78A126* in biosynthetic flavonoid pathways, and they are natural products that could protect plants through scavenging ROS under drought/NaCl stresses. Meanwhile, *CYP74B4* and *CYP94A52* may be involved in oxylipin pathway and *CYP83D3* in glucosinolates pathway, and the accumulation of these compounds could avoid water loss by stomatal closure to improve tolerance under drought/NaCl stress. In addition, *CYP704G6* may participate in fatty acid biosynthesis and may affect flower development under drought stress, and *CYP71A31* and *CYP71AU56* may be involved in IAA biosynthesis to improve plant tolerance under drought stress. In summary, the identification of these *MtP450* genes provided clues for function characterization and their possible application in stress-related breeding in *Medicago*. Nevertheless, these *MtP450* genes encode unique enzymes that individually catalyze certain reactions for a group of specific compounds, and their biochemical and genetic functions require further characterization.

## 4. Materials and Methods

### 4.1. Identification of Cytochrome P450 Genes in the M. truncatula Genome

*P450* genes that were previously identified in *M. truncatula* were retrieved from the Cytochrome P450 Homepage website (http://drnelson.uthsc.edu/CytochromeP450.html, access time: 20 June 2015). Additional full-length *MtP450* genes were identified based on their domain predictions from Pfam (https://pfam.xfam.org/, access time: 10 April 2020) [67] and Panther (http://www.pantherdb.org/about.jsp, access time: 10 April 2020) [68]. The present results were consolidated with previously identified P450s, and the pseudogenes were listed as on the P450 homepage website.

### 4.2. Sequence Analyses, Structural Characterization and Phylogenetic Tree Construction

Protein sequences of *MtP450* genes were obtained based on Phytozome v12.1. Physical and chemical properties of MtP450s proteins, including their number of amino acids, molecular weights and theoretical isoelectric points (pI), were calculated by using online ProtParam tools (https://web.expasy.org/protparam/, access time: 12 April 2020). The protein subcellular localization of MtP450s were predicted by the ProtComp v.9.0 server (http://linux1.softberry.com/berry.phtml?topic=protcomppl&group=programs&subgroup=proloc, access time: 15 April 2020). Sequence alignments were analyzed by using ClustalX (http://www.jalview.org/Web_Installers/install.htm, access time: 12 May 2020). Conserved motifs of MtP450s proteins were identified by using the MEME program (MEME-Suite version 5.1.0, http://meme-suite.org/, access time: 15 May 2020) with default settings [69], except that the motif number was set to 10 and the width of minimum and maximum motif were set as 10 and 200, respectively. The chromosomal localization of *MtP450* genes were acquired from the website for the *M. truncatula* genome. The exon–intron positions and distributions of intron phases were analyzed through Amazing Optional Gene Viewer software [70].

To investigate the phylogenetic relationship among MtP450s, their protein sequences were aligned using ClustalX and MEGA-X. Firstly, multiple sequence alignment were performed using the ClustalX with default parameters. Then, an unrooted neighbor-joining phylogenetic tree was constructed based on the neighbor-joining method, with the robustness of tree topology assessed by 1000 bootstrap replicates using the MEGA-X software [71].

### 4.3. Chromosomal Localization and Gene Duplication Events

The gene duplication events were analyzed using multiple Collinearity Scan toolkit (MCScanX) with default parameters [72]. All *MtP450* genes were mapped to 8 chromosomes and 10 scaffolds based on their physical location (GFF3 formatted file), followed by the analysis on their intraspecific synteny relationship via TBtools (https://github.com/CJ-Chen/TBtools, access time: 20 July 2020) using Amazing Gene Location. To exhibit the interspecific synteny relationship between *M. truncatula*, *Arabidopsis* and rice, the syntenic maps were constructed using the Dual Synteny Plotter software; non-synonymous (Ka) and synonymous (Ks) values of the *MtP450* homologous gene pairs were calculated using Simple Ka/Ks Calculator software [73].

### 4.4. Analysis of the Cis-Acting Elements in Promoter Region of MtP450 Genes

The 2 kb sequences upstream individual *MtP450* genes were uploaded to PlantCARE (http://bioinformatics.psb.ugent.be/webtools/plantcare/html/, access time: 20 April 2020) [74] and analyzed to predict the *cis* elements in their promoter regions.

### 4.5. Microarray-Based Gene Expression Profiling

Gene expression data were originally downloaded from the Gene Chip data of the *M. truncatula* Gene Expression Atlas (https://mtgea.noble.org/v3/, access time: 10 September 2015), and the information is currently available at ArrayExpress Database (https://www.ebi.ac.uk/arrayexpress/, access time: 10 September 2015) under accession number E-MEXP-1097 [13], E-MTAB-2681 [75], E-GEOD-13907 and E-GEOD-13921 [76]. The selected genechip data covered all its major organ systems (roots, stems, leaves, petioles, vegetative buds, flowers, seed pods, and seeds of 24 days after pollination (24-dap seeds), and plants subjected to drought/NaCl stresses. Amazing HeatMap software was used to generate the heatmap by using the TB tools.

### 4.6. Weighted Gene Co-Expression Network Analysis (WGCNA)

Gene co-expression networks were constructed using the WGCNA package. The selected genechip data for 204 *MtP450* genes subjected to drought/NaCl stresses were imported into WGCNA. A matrix of pair-wise SCCs between all pairs of genes was generated and transformed into an adjacency matrix. The modules were obtained using the automatic network construction function blockwiseModules with default settings, except that the settings for TOMType is unsigned, minModuleSize is 30, and mergeCutHeight is 0.25. The eigengene value was calculated for each module, which was used to determine the association analysis of expression pattern response to each treatment. Network visualization for each module was performed using the Cytoscape software version 3.6.1 with a cut off weight parameter of 0.2 [77].

### 4.7. Plant Materials and qPCR Validation

The seeds of *M. truncatula* (cv. Jemalong A17) were originally obtained from the Noble Research Institute and maintained in our group. The seeds of *M. truncatula* were germinated and transferred into MS liquid medium, then grown in a growth chamber with 16 h light/8 h dark. After the third leaf was fully expanded, plants were respectively supplied with PEG/NaCl at final concentration of 0.1 mM. The whole plants were collected at 0 h (as control), 3 h, 24 h, 48 h, and 72 h after treatments, and immediately frozen in liquid nitrogen and stored at −80°C for subsequent analysis.

Total RNAs were extracted with the Eastep^®^ Super total RNA Extraction kit (Promega, Shanghai, China) according to the manufacturer’s instructions. The first-strand cDNA was synthesized by using Trans^®^ Script One-Step gDNA Removal and cDNA Synthesis SuperMix (TransGen Biotech, Beijing, China). qPCRs were performed with a 2× RealStar Green Fast Mixture (GeneStar, Shanghai, China) on a ABI Q7 QuantStudio^TM^ Real-Time PCR Detection System (Applied Biosystems, Waltham, MA, USA). *Actin* gene was used as housekeeping gene. PCRs were performed with the following program: 94 °C for 30 s, followed by 40 cycles of 94 °C, 5 s, 60 °C, 34 s. Melting Curve analysis was performed by using ABI Q7 QuantStudio^TM^ Real-Time PCR Software with the qPCR data. The transcript levels of each gene were determined by relative quantification using the 2^−ΔΔCt^ method and normalized with *actin* gene as a reference [78]. Data are average of three independent biological samples ±SE and vertical bars indicate standard deviation. Student’s t test was used to compare the difference between two treatments (*n* = 3, * *p* < 0.05, ** *p* < 0.01). The primer sequences used in this study were shown in Table S8.

## Data Availability

All data in the present study are available in the public database as referred in the Material and Methods section.

## Supplementary Materials

The following are available online at https://www.mdpi.com/article/10.3390/ijms22189957/s1.
