# Supplementary material for: Genome-Wide Identification and Analyses of Drought/Salt-Responsive Cytochrome P450 Genes in Medicago truncatula
Source: Int J Mol Sci. 2021 Sep 15;22(18):9957. doi: 10.3390/ijms22189957 (PMC8467197; doi:10.3390/ijms22189957)
Supplement: Supplementary file 1 [file ijms-22-09957-s001.zip › Supporting information 20210820.pdf]

Supporting Information

Table S1 Locations of the 444 *MtP450* genes and the proprieties of the predicted 346 MtP450 proteins.

Clan sand numbers	Gene name	Gene ID	Locus	Aa	MW	Theoretical pI	Predicted destination
**CYP51 (1)**	CYP51G1	Medtr8g006450	577582..581419	489	55.63	7.24	Plasma membrane
**CYP701(1)**	CYP701A17	Medtr2g105360	45424314..45427720	502	57.76	7.00	Membrane bound Chloroplast
**CYP703(1)**	CYP703A8	Medtr7g087520	34077176..34078819	242	27.63	5.36	Endoplasmic reticulum
**CYP704(9)**	CYP704B11	Medtr5g019140	7235136..7237948	521	59.24	8.33	Plasma membrane
	CYP704B38	Medtr4g119660	49590462..49592870	531	61.31	9.26	Plasma membrane
	CYP704G16	Medtr8g035860	13113098..13115707	516	59.46	8.85	Plasma membrane
	CYP704G2	Medtr8g035820	13102355..13106171	510	58.57	7.17	Plasma membrane
	CYP704G3	Medtr8g035810	13094804..13100514	510	58.60	7.96	Plasma membrane
	CYP704G5	Medtr8g035780	13076689..13081002	511	58.84	7.58	Plasma membrane
	CYP704G6	Medtr8g035760	13068560..13073043	515	59.59	9.03	Plasma membrane
	CYP704G7v2	Medtr8g035680	13020800..13024338	512	58.85	8.98	Plasma membrane
	CYP704G9	Medtr1g102220	46164562..46167651	513	59.05	9.02	Plasma membrane
**CYP706(2)**	CYP706A12	Medtr4g102280	42375953..42379136	508	58.55	8.56	Plasma membrane
	CYP706A19	Medtr4g102310	42392718..42394804	516	58.15	6.00	Plasma membrane
**CYP707(6)**	CYP707A105	Medtr1g037580	13909295..13915912	510	57.43	6.58	Plasma membrane
	CYP707A106	Medtr4g086040	33657251..33660334	478	54.89	9.49	Endoplasmic reticulum
	CYP707A107	Medtr4g086130	33698058..33700908	473	54.52	9.37	Endoplasmic reticulum
	CYP707A17	Medtr5g025610	10422423..10425348	469	53.94	9.32	Endoplasmic reticulum
	CYP707A18	Medtr1g019410	5858845..5862486	464	53.10	8.89	Endoplasmic reticulum
	CYP707A19	Medtr8g072260	30521508..30524747	482	55.22	9.41	Endoplasmic reticulum
**CYP709(1)**	CYP709B4	Medtr1g085680	38291709..38299312	466	53.21	9.00	Endoplasmic reticulum
**CYP710(1)**	CYP710A15	Medtr2g019640	6454155..6456401	518	59.46	8.99	Plasma membrane
**CYP711(2)**	CYP711A10	Medtr1g015860	4089186..4092561	515	58.53	7.22	Plasma membrane
	CYP711A12	Medtr3g104560	48182777..48184409	529	59.77	9.21	Plasma membrane
**CYP712(2)**	CYP712B1	Medtr7g092600	36727608..36730862	541	61.34	8.12	Plasma membrane
	CYP712D5	Medtr8g036185	13370006..13372792	513	58.34	8.32	Plasma membrane
**CYP714(6)**	CYP714A4	Medtr3g093530	42738174..42746014	524	59.07	6.09	Plasma membrane
	CYP714E1	Medtr7g055793	19205156..19208490	524	59.59	8.70	Endoplasmic reticulum
	CYP714E22	Medtr8g469360	25195572..25197962	524	59.47	8.92	Endoplasmic reticulum
	CYP714E23	Medtr8g008530	1760305..1763493	424	47.64	8.40	Endoplasmic reticulum
	CYP714E7	Medtr0147s0030	8011..11113	519	58.81	9.17	Endoplasmic reticulum
	CYP714H6	Medtr2g061200	25927980..25932020	531	60.18	8.59	Endoplasmic reticulum
**CYP715(5)**	CYP715A26	Medtr1g101040	45410521..45413445	535	61.09	9.05	Endoplasmic reticulum
	CYP715A27	Medtr1g101050	45419830..45422711	516	58.97	8.30	Plasma membrane
	CYP715A28	Medtr0025s0110	63351..67418	512	58.58	8.52	Plasma membrane
	CYP715A29	Medtr1g069200	29799588..29803592	526	60.61	9.02	Plasma membrane
	CYP715A4	Medtr6g072490	26835021..26837569	512	58.81	8.96	Plasma membrane

**CYP716(2)**	CYP716A12	Medtr8g100135	42436911..42441542	514	58.76	9.20	Extracellular (Secreted)
	CYP716G1	Medtr8g089190	37053837..37055680	479	54.71	8.88	Plasma membrane
**CYP718(1)**	CYP718A8	Medtr5g091760	40041438..40043223	445	50.59	8.99	Plasma membrane
**CYP71(59)**	CYP71A29	Medtr3g077460	34792157..34793856	479	55.35	9.29	Endoplasmic reticulum
	CYP71A30	Medtr3g076560	34416428..34418100	392	45.15	8.82	Plasma membrane
	CYP71A31	Medtr4g104640	43341256..43344509	521	59.59	7.52	Plasma membrane
	CYP71A60	Medtr3g076550	34413124..34415596	521	59.73	7.13	Plasma membrane
	CYP71A61	Medtr4g104660	43359064..43361426	477	53.90	6.53	Plasma membrane
	CYP71AP16	Medtr3g076530	34404975..34406754	516	58.98	8.84	Plasma membrane
	CYP71AU56	Medtr4g089020	35641126..35643058	509	57.83	6.06	Plasma membrane
	CYP71AU57	Medtr4g089025	35645980..35650524	497	56.32	8.34	Plasma membrane
	CYP71AU58	Medtr4g089030	35655408..35657204	522	59.55	8.01	Plasma membrane
	CYP71AU59	Medtr4g089055	35676374..35678680	521	59.22	5.96	Plasma membrane
	CYP71AU60	Medtr4g104540	43266869..43268909	521	59.07	6.33	Plasma membrane
	CYP71AU61	Medtr4g104550	43273970..43275874	431	48.89	6.63	Plasma membrane
	CYP71AU62	Medtr4g104610	43307305..43311594	495	56.07	9.14	Plasma membrane
	CYP71AU7	Medtr0022s0290	90566..93099	500	56.57	6.72	Plasma membrane
	CYP71D356	Medtr1g035140	12636378..12639032	518	58.90	6.45	Plasma membrane
	CYP71D417	Medtr4g048580	17248200..17250064	505	56.87	8.65	Plasma membrane
	CYP71D418	Medtr2g010530	2431589..2433884	502	57.57	8.71	Plasma membrane
	CYP71D420	Medtr8g466180	23610015..23611766	500	57.10	8.68	Plasma membrane
	CYP71D421	Medtr0554s0020	4993..7144	507	58.09	8.56	Plasma membrane
	CYP71D422	Medtr3g058210	23016585..23018850	508	57.99	8.62	Plasma membrane
	CYP71D423	Medtr3g058220	23023886..23026030	509	57.84	8.75	Plasma membrane
	CYP71D424	Medtr3g058230	23032512..23034160	507	57.98	8.26	Plasma membrane
	CYP71D425	Medtr3g058240	23035968..23037585	429	49.03	6.24	Plasma membrane
	CYP71D426	Medtr3g058250	23038848..23040564	473	54.05	8.63	Plasma membrane
	CYP71D427	Medtr3g058380	23139844..23141550	504	57.77	8.44	Plasma membrane
	CYP71D428	Medtr3g058390	23145718..23147439	427	48.97	6.20	Plasma membrane
	CYP71D429	Medtr3g058400	23148325..23150526	507	58.09	7.57	Plasma membrane
	CYP71D430	Medtr6g462640	21779283..21781458	508	57.87	8.22	Plasma membrane
	CYP71D431	Medtr4g133750	55954391..55956711	509	58.20	8.70	Plasma membrane
	CYP71D432	Medtr4g133760	55958245..55959898	503	57.20	9.01	Plasma membrane
	CYP71D433	Medtr6g034965	12265825..12268663	389	44.10	7.09	Plasma membrane
	CYP71D434	Medtr6g035100	12425022..12426995	500	57.42	8.90	Plasma membrane
	CYP71D435	Medtr6g035115	12438102..12439853	500	56.97	8.64	Plasma membrane
	CYP71D436	Medtr0059s0170	61740..63732	503	56.94	8.60	Plasma membrane
	CYP71D437	Medtr1g035190	12609514..12612847	507	57.62	8.28	Plasma membrane
	CYP71D59	Medtr5g094520	41314159..41317550	492	55.17	7.62	Plasma membrane
	CYP71D61	Medtr5g094540	41323569..41325528	533	61.25	8.50	Plasma membrane
	CYP71D62	Medtr5g094550	41327441..41329763	502	57.72	8.66	Plasma membrane
	CYP71D63	Medtr5g094560	41331507..41333381	502	57.97	7.00	Plasma membrane
	CYP71D64	Medtr5g094570	41336436..41338326	502	57.96	8.27	Plasma membrane

	CYP71D66	Medtr5g094660	41365761..41367473	502	57.45	8.63	Plasma membrane
	CYP71D69v1	Medtr3g057800	22854929..22858976	501	57.55	9.20	Plasma membrane
	CYP71D70	Medtr3g057860	22901428..22905404	501	56.94	8.56	Plasma membrane
	CYP71D72	Medtr3g057900	22913843..22917325	506	57.58	8.63	Plasma membrane
	CYP71D73	Medtr3g057910	22918706..22920775	499	56.95	8.55	Plasma membrane
	CYP71D74	Medtr3g057970	22931720..22934239	509	57.62	8.84	Plasma membrane
	CYP71D75	Medtr3g057980	22939282..22941877	493	56.29	8.94	Plasma membrane
	CYP71D77	Medtr3g058000	22946208..22947981	498	56.67	6.45	Plasma membrane
	CYP71D78	Medtr3g058060	22971128..22973864	509	58.36	8.53	Plasma membrane
	CYP71D79	Medtr3g058110	22985915..22987741	600	68.52	7.10	Plasma membrane
	CYP71D81	Medtr5g073320	31224520..31226361	511	58.26	8.65	Plasma membrane
	CYP71D82	Medtr5g073250	31187579..31189290	503	57.51	7.66	Plasma membrane
	CYP71D85	Medtr5g018980	7129927..7132226	502	57.56	8.55	Plasma membrane
	CYP71D86	Medtr5g018990	7140287..7141971	507	57.35	8.87	Plasma membrane
	CYP71D87	Medtr5g019010	7152073..7154888	506	57.95	9.20	Plasma membrane
	CYP71D89	Medtr5g095260	41645591..41647565	505	57.75	7.69	Plasma membrane
	CYP71D90	Medtr5g095290	41652786..41655432	503	57.33	9.09	Plasma membrane
	CYP71D92	Medtr6g039630	14307043..14307879	502	57.02	8.91	Plasma membrane
	CYP71D93	Medtr1g442730	15884513..15886275	278	32.15	9.67	Plasma membrane
**CYP720(1)**	CYP720A1	Medtr6g069000	24809118..24812860	504	57.25	8.28	Plasma membrane
**CYP721(2)**	CYP721A11	Medtr7g098550	39448422..39451869	452	51.73	8.05	Plasma membrane
	CYP721A40	Medtr1g022040	6789887..6793507	507	58.49	8.80	Endoplasmic reticulum
**CYP722(2)**	CYP722A1	Medtr3g112120	52474500..52477877	503	58.11	9.16	Plasma membrane
	CYP722C1	Medtr8g076940	32650395..32654772	493	56.23	9.27	Plasma membrane
**CYP727(1)**	CYP727B11	Medtr3g012320	3358725..3361617	492	56.62	8.96	Plasma membrane
**CYP728(3)**	CYP728B29	Medtr5g092150	40242651..40245124	195	21.69	6.83	Extracellular (Secreted)
	CYP728H6	Medtr3g467130	27669661..27672771	474	53.58	9.56	Plasma membrane
	CYP728H7	Medtr3g467140	27673375..27677774	481	55.65	8.80	Plasma membrane
**CYP729(3)**	CYP729A25	Medtr0045s0070	30625..36273	482	55.23	8.93	Endoplasmic reticulum
	CYP729A26	Medtr0045s0080	38431..43007	477	54.28	9.28	Endoplasmic reticulum
	CYP729A5	Medtr0045s0060	22594..28397	410	47.37	9.49	Endoplasmic reticulum
**CYP72(15)**	CYP72A336	Medtr2g072250	30381481..30385408	477	54.89	9.22	Endoplasmic reticulum
	CYP72A337	Medtr2g072270	30400470..30405069	516	59.41	8.96	Plasma membrane
	CYP72A338	Medtr2g072310	30424007..30425561	523	60.25	9.04	Plasma membrane
	CYP72A340	Medtr2g072340	30434253..30437998	353	40.03	5.90	Plasma membrane
	CYP72A341	Medtr2g072400	30472571..30477011	518	59.14	8.07	Plasma membrane
	CYP72A59v1	Medtr2g072380	30465654..30469833	518	59.38	9.25	Plasma membrane
	CYP72A61v1	Medtr4g031820	11016195..11019902	493	56.35	8.44	Plasma membrane
	CYP72A62v1	Medtr8g042060	15865215..15868880	528	60.07	9.19	Plasma membrane
	CYP72A64	Medtr8g042020	15851409..15854768	524	59.64	9.21	Plasma membrane
	CYP72A65v1	Medtr8g042000	15844075..15847819	523	59.71	8.70	Plasma membrane
	CYP72A66	Medtr2g072260	30390835..30396174	524	59.39	8.74	Plasma membrane
	CYP72A67v1	Medtr2g023680	8378417..8382727	513	58.96	8.88	Plasma membrane

	CYP72A68v1	Medtr2g055470	23782822..23785860	520	59.88	8.97	Plasma membrane
	CYP72A68v2	Medtr2g055430	23765693..23769179	520	59.90	9.14	Plasma membrane
	CYP72A70	Medtr5g095230	41626489..41632042	520	59.81	8.91	Plasma membrane
**CYP733(1)**	CYP733A1	Medtr6g034940	12228075..12232530	516	59.69	9.13	Plasma membrane
**CYP734(1)**	CYP734A12	Medtr4g113650	46750295..46754779	481	55.13	9.05	Plasma membrane
**CYP735(2)**	CYP735A10	Medtr6g017325	6970322..6975980	530	60.42	9.22	Plasma membrane
	CYP735A28	Medtr7g006900	1266094..1270905	509	57.57	8.86	Plasma membrane
**CYP736(15)**	CYP736A104	Medtr2g065360	27411723..27413733	474	53.92	9.06	Plasma membrane
	CYP736A129	Medtr0638s0020	4522..7150	473	53.48	6.34	Plasma membrane
	CYP736A13	Medtr6g084770	31889040..31891162	430	48.66	6.29	Plasma membrane
	CYP736A130	Medtr5g070850	29943543..29945561	508	58.42	8.70	Plasma membrane
	CYP736A131	Medtr5g070830	29950460..29954250	512	58.27	8.48	Plasma membrane
	CYP736A132a	Medtr5g070690	29962646..29965777	622	72.38	5.14	Plasma membrane
	CYP736A132b	Medtr5g070800	30009280..30012023	510	58.35	7.63	Plasma membrane
	CYP736A133	Medtr5g470730	29990374..29995754	510	58.35	7.63	Plasma membrane
	CYP736A134	Medtr5g070710	29998924..30001861	427	48.47	8.72	Plasma membrane
	CYP736A135	Medtr5g070700	30005455..30007795	508	57.85	6.49	Plasma membrane
	CYP736A136	Medtr5g070920	30043134..30045728	511	58.45	7.23	Plasma membrane
	CYP736A139	Medtr8g091680	38257869..38260373	510	57.90	8.53	Plasma membrane
	CYP736A140	Medtr8g091690	38261626..38263335	457	52.27	7.17	Plasma membrane
	CYP736A142	Medtr8g104080	43805389..43807381	477	54.11	8.38	Plasma membrane
	CYP736A142	Medtr8g104100	43812601..43813404	283	31.61	9.34	Plasma membrane
**CYP73(2)**	CYP73A121	Medtr1g111240	50239925..50242309	267	30.55	6.21	Plasma membrane
	CYP73A3	Medtr5g075450	32080406..32084522	537	61.52	8.61	Plasma membrane
**CYP74(5)**	CYP74A1	Medtr1g021652	6532322..6534417	506	58.17	9.10	Plasma membrane
	CYP74A37	Medtr3g111530	52111958..52113811	524	59.44	9.10	Membrane bound Chloroplast
	CYP74B4v1	Medtr2g087890	37001502..37006215	559	62.90	9.10	Cytoplasmic
	CYP74C12	Medtr1g034320	12490318..12493204	480	53.75	8.18	Membrane bound Chloroplast
	CYP74C13	Medtr4g068550	25731961..25733983	485	54.54	8.51	Cytoplasmic
**CYP75(6)**	CYP75A59	Medtr3g436540	12278462..12281860	482	53.93	6.16	Membrane bound Chloroplast
	CYP75A60	Medtr3g436390	12221643..12226531	515	58.38	9.28	Plasma membrane
	CYP75B82	Medtr3g024520	7619578..7624416	506	56.77	9.06	Plasma membrane
	CYP75B83	Medtr3g025230	7659217..7664309	516	57.80	8.05	Plasma membrane
	CYP75B85	Medtr3g025260	7674967..7683298	516	57.77	8.31	Plasma membrane
	CYP75B86	Medtr4g109470	45477291..45481844	516	57.83	6.74	Plasma membrane
**CYP76(25)**	CYP76A39	Medtr7g103450	41858984..41861868	510	56.98	6.98	Plasma membrane
	CYP76D2	Medtr5g056720	23342701..23347011	519	60.04	8.40	Plasma membrane
	CYP76E1	Medtr7g075600	28318943..28322524	425	48.70	8.13	Plasma membrane
	CYP76E19	Medtr7g075390	28295531..28297440	503	56.69	8.34	Plasma membrane
	CYP76E2	Medtr7g075540	28287872..28291257	505	57.09	7.58	Plasma membrane
	CYP76E21	Medtr7g075580	28306169..28310820	503	56.62	8.67	Plasma membrane
	CYP76E22	Medtr7g075610	28328062..28332016	503	56.69	8.00	Plasma membrane
	CYP76F70	Medtr8g046210	18091825..18094727	504	56.67	8.68	Plasma membrane

	CYP76F71	Medtr8g046240	18101780..18104684	509	57.43	6.00	Plasma membrane
	CYP76F72	Medtr4g078110	29972262..29975536	509	57.34	6.00	Plasma membrane
	CYP76F73	Medtr1g037370	13789447..13793333	479	53.76	9.39	Plasma membrane
	CYP76F74	Medtr1g031700	11106226..11107837	496	55.93	8.94	Plasma membrane
	CYP76F76	Medtr1g103670	46926221..46928257	499	55.96	8.66	Plasma membrane
	CYP76F77	Medtr1g103680	46933000..46934958	499	56.01	8.55	Plasma membrane
	CYP76G18	Medtr1g116890	52795806..52798189	500	56.08	9.10	Plasma membrane
	CYP76X13	Medtr1g091003	41118878..41120521	522	58.88	8.34	Plasma membrane
	CYP76X14	Medtr1g091010	41127541..41129848	485	55.16	6.86	Plasma membrane
	CYP76X15	Medtr1g055325	24466877..24469147	489	55.76	7.70	Plasma membrane
	CYP76X16	Medtr2g073420	31129442..31132001	507	57.44	6.81	Plasma membrane
	CYP76X17	Medtr2g073440	31135127..31137701	500	56.61	8.16	Plasma membrane
	CYP76X2	Medtr1g091023	41142961..41145287	469	53.04	8.73	Plasma membrane
	CYP76X3	Medtr5g007450	1363092..1365323	489	55.59	8.56	Plasma membrane
	CYP76X4	Medtr5g007460	1368811..1370704	500	56.55	8.42	Endoplasmic reticulum
	CYP76X5	Medtr5g007550	1406233..1408464	479	54.33	6.54	Plasma membrane
	CYP76Y14	Medtr1g097240	43861808..43864496	500	56.50	8.60	Plasma membrane
**CYP77(2)**	CYP77A12	Medtr1g492820	41622203..41624045	498	56.34	6.98	Plasma membrane
	CYP77B5	Medtr4g131830	55015440..55017302	520	58.77	9.18	Endoplasmic reticulum
**CYP78(4)**	CYP78A124	Medtr1g097220	43849660..43852676	508	57.74	8.85	Endoplasmic reticulum
	CYP78A125	Medtr5g045250	19855600..19857686	530	59.98	9.05	Plasma membrane
	CYP78A126	Medtr8g080520	34636764..34638483	520	58.92	6.61	Plasma membrane
	CYP78A29	Medtr8g027040	9421677..9424147	530	59.70	8.99	Plasma membrane
**CYP79(8)**	CYP79D12	Medtr8g073960	31274392..31276437	546	61.34	7.62	Plasma membrane
	CYP79D13	Medtr8g073950	31268723..31270653	531	60.30	8.96	Endoplasmic reticulum
	CYP79D14	Medtr8g076390	32362541..32364526	527	59.89	7.95	Endoplasmic reticulum
	CYP79D41	Medtr3g465300	26408512..26411886	530	60.37	8.37	Endoplasmic reticulum
	CYP79D42	Medtr8g076372	32345873..32347757	531	60.50	8.97	Endoplasmic reticulum
	CYP79D43	Medtr3g467200	27697546..27700034	518	58.81	8.72	Endoplasmic reticulum
	CYP79D44	Medtr3g467220	27710421..27714552	533	60.71	8.78	Endoplasmic reticulum
	CYP79D45	Medtr3g467290	27745660..27747404	528	60.49	8.94	Endoplasmic reticulum
**CYP81(9)**	CYP81E10	Medtr5g016440	5892570..5894341	539	61.30	8.69	Endoplasmic reticulum
	CYP81E57	Medtr2g437840	14944550..14946740	495	56.84	8.77	Plasma membrane
	CYP81E58	Medtr4g095050	39583537..39587364	500	58.05	8.96	Plasma membrane
	CYP81E59	Medtr4g094772	38931126..38934189	509	57.80	9.06	Plasma membrane
	CYP81E60	Medtr4g035330	12151050..12156251	494	56.37	9.00	Plasma membrane
	CYP81E7	Medtr4g094775	38953546..38956603	491	56.44	8.74	Plasma membrane
	CYP81E8v2	Medtr5g016410	5883760..5886827	498	57.64	8.95	Plasma membrane
	CYP81E9	Medtr2g437880	14956039..14961072	499	57.30	9.10	Plasma membrane
	CYP81X3	Medtr4g077210	29582833..29587271	503	57.45	8.54	Plasma membrane
**CYP82(19)**	CYP82A11	Medtr6g008640	2361289..2363666	509	58.27	7.93	Plasma membrane
	CYP82A12	Medtr6g008650	2365823..2369279	529	60.10	8.66	Plasma membrane
	CYP82A13	Medtr4g018800	5804032..5806388	531	60.57	8.26	Plasma membrane

	CYP82A14	Medtr6g042540	14711142..14714952	532	60.26	8.00	Plasma membrane
	CYP82A15	Medtr6g042610	14753204..14755390	521	59.54	8.28	Plasma membrane
	CYP82A16	Medtr3g014880	4302926..4306458	525	59.92	8.08	Plasma membrane
	CYP82A5	Medtr6g008430	2261074..2264314	532	60.26	6.10	Plasma membrane
	CYP82A6	Medtr6g008500	2294908..2297955	524	59.42	8.69	Plasma membrane
	CYP82A7	Medtr6g008530	2305547..2307779	541	61.64	7.63	Plasma membrane
	CYP82A8	Medtr6g008600	2337903..2340449	535	60.51	6.74	Plasma membrane
	CYP82A9	Medtr6g008620	2348811..2352387	533	60.37	7.98	Plasma membrane
	CYP82C50	Medtr5g018480	6898409..6899371	531	60.10	8.44	Plasma membrane
	CYP82D47	Medtr2g450040	22061730..22065853	320	36.35	8.72	Plasma membrane
	CYP82D94	Medtr4g088400	35019222..35027135	524	59.08	7.27	Plasma membrane
	CYP82D96	Medtr4g088415	35035590..35038803	526	59.69	7.25	Plasma membrane
	CYP82D97	Medtr4g088425	35043854..35048424	376	43.01	9.53	Plasma membrane
	CYP82J7	Medtr6g022450	7841391..7843353	510	57.39	7.63	Plasma membrane
	CYP82J8	Medtr8g012590	3657106..3659102	516	58.83	7.74	Plasma membrane
	CYP82L14	Medtr8g012585	3651861..3655164	506	58.01	6.19	Plasma membrane
**CYP83(18)**	CYP83D3	Medtr1g023730	7633868..7637366	458	52.41	8.05	Plasma membrane
	CYP83D4	Medtr1g023720	7626885..7630409	514	58.99	8.31	Plasma membrane
	CYP83D5	Medtr1g023700	7622290..7624049	514	58.96	7.67	Plasma membrane
	CYP83E10	Medtr7g012860	3629642..3632170	515	59.03	6.45	Plasma membrane
	CYP83E10	Medtr0287s0060	14014..16140	501	57.30	7.33	Plasma membrane
	CYP83E11	Medtr5g045770	20073801..20076195	501	57.30	7.33	Plasma membrane
	CYP83E39	Medtr4g025950	8904616..8906158	497	57.36	8.52	Plasma membrane
	CYP83E40	Medtr4g025970	8914247..8917002	311	36.29	8.71	Plasma membrane
	CYP83E41	Medtr4g026030	8952646..8955502	498	57.37	8.53	Plasma membrane
	CYP83E42	Medtr4g026050	8965558..8972823	345	39.48	5.00	Plasma membrane
	CYP83E43	Medtr4g026070	8975279..8977558	497	57.16	7.99	Plasma membrane
	CYP83E44	Medtr4g026200	9026952..9031194	310	36.22	8.93	Plasma membrane
	CYP83E45	Medtr5g023680	9399854..9401775	497	56.75	6.82	Plasma membrane
	CYP83E8	Medtr4g025980	8929046..8930923	473	54.36	6.88	Plasma membrane
	CYP83E9	Medtr7g012330	3619569..3621677	497	57.21	8.08	Plasma membrane
	CYP83G1v1a	Medtr5g072930	31038885..31040934	500	56.98	8.59	Plasma membrane
	CYP83G1v1b	Medtr5g072980	31052311..31054387	506	57.75	8.49	Plasma membrane
	CYP83G2	Medtr5g073020	31071402..31074005	506	57.75	8.49	Plasma membrane
**CYP84(3)**	CYP84A17	Medtr5g021390	8259083..8262495	506	58.21	7.97	Plasma membrane
	CYP84A18	Medtr8g076290	32312590..32316653	519	58.50	5.90	Plasma membrane
	CYP84A64	Medtr4g071710	27144853..27148498	510	57.48	6.11	Plasma membrane
**CYP85(3)**	CYP85A1	Medtr6g015910	5587861..5591895	518	58.45	6.36	Plasma membrane
	CYP85A32	Medtr3g007320	973176..976261	464	53.55	9.25	Plasma membrane
	CYP85A33	Medtr7g016040	5062210..5067832	464	53.54	9.29	Plasma membrane
**CYP86(8)**	CYP86A23	Medtr5g070010	29666985..29669859	463	53.54	9.26	Plasma membrane
	CYP86A24	Medtr8g030590	11313150..11316076	510	58.40	9.15	Plasma membrane
	CYP86A93	Medtr8g098435	41064632..41066861	540	61.21	8.43	Plasma membrane

	CYP86A94	Medtr3g463060	25242873..25244861	517	59.11	8.58	Plasma membrane
	CYP86B18	Medtr1g037550	13886339..13889214	510	58.34	9.15	Plasma membrane
	CYP86B19	Medtr1g037560	13895668..13898642	559	64.81	8.33	Plasma membrane
	CYP86B20	Medtr7g406990	1033660..1035589	555	64.55	8.83	Plasma membrane
	CYP86B6	Medtr4g075110	28665652..28667944	446	51.86	8.49	Plasma membrane
**CYP87(1)**	CYP87A9	Medtr5g010750	2946786..2949176	559	64.49	8.71	Plasma membrane
**CYP88(10)**	CYP88A13	Medtr5g014240	4746397..4752048	483	55.39	8.95	Plasma membrane
	CYP88A14	Medtr5g014250	4756107..4759334	490	56.53	9.07	Endoplasmic reticulum
	CYP88A54	Medtr2g031930	12112690..12119126	491	56.58	9.21	Endoplasmic reticulum
	CYP88A55	Medtr2g031920	12104335..12107613	491	56.76	9.08	Endoplasmic reticulum
	CYP88D1	Medtr4g005230	124806..131589	490	56.62	9.26	Endoplasmic reticulum
	CYP88D10	Medtr1g032950	11799270..11805170	487	56.19	8.40	Endoplasmic reticulum
	CYP88D11	Medtr1g032980	11814838..11820504	477	55.05	8.61	Endoplasmic reticulum
	CYP88D2	Medtr8g012970	3956880..3962052	399	45.84	9.02	Endoplasmic reticulum
	CYP88D3	Medtr8g089800	37431289..37437039	489	56.60	9.07	Endoplasmic reticulum
	CYP88D9	Medtr4g029690	10303062..10306339	492	56.49	9.18	Endoplasmic reticulum
**CYP89(12)**	CYP89A124	Medtr8g098565	41126000..41127523	327	37.82	9.04	Endoplasmic reticulum
	CYP89A125	Medtr8g063260	26493585..26495108	507	58.48	8.29	Endoplasmic reticulum
	CYP89A126	Medtr8g063280	26505629..26507255	507	58.71	8.90	Endoplasmic reticulum
	CYP89A127	Medtr5g026520	10901472..10903004	499	57.82	6.67	Endoplasmic reticulum
	CYP89A128	Medtr5g034900	15176190..15177746	510	59.33	8.83	Endoplasmic reticulum
	CYP89A28	Medtr2g010250	2292738..2294592	518	60.31	8.56	Endoplasmic reticulum
	CYP89A29	Medtr2g010280	2302389..2303938	506	58.52	8.13	Endoplasmic reticulum
	CYP89A30	Medtr2g010290	2309007..2310762	504	58.40	6.96	Endoplasmic reticulum
	CYP89A31	Medtr2g010300	2320125..2321717	533	61.47	9.15	Endoplasmic reticulum
	CYP89A32	Medtr2g010320	2325979..2327493	490	56.61	6.56	Endoplasmic reticulum
	CYP89A33	Medtr2g010330	2329544..2331662	504	58.32	8.52	Endoplasmic reticulum
	CYP89A34	Medtr2g010380	2349982..2351514	508	58.30	8.56	Endoplasmic reticulum
**CYP90(7)**	CYP90A14	Medtr3g070380	31551294..31557836	510	58.84	8.76	Endoplasmic reticulum
	CYP90A40	Medtr5g082520	35472249..35479791	490	56.27	9.10	Plasma membrane
	CYP90B10	Medtr8g077810	33099721..33108214	472	54.25	9.56	Plasma membrane
	CYP90B11	Medtr5g020020	7617615..7623052	508	58.35	7.58	Plasma membrane
	CYP90C20	Medtr5g021220	8157328..8163208	483	55.19	8.84	Plasma membrane
	CYP90C4	Medtr8g076510	32416342..32428226	491	56.25	9.14	Endoplasmic reticulum
	CYP90D27	Medtr8g016180	5411276..5415164	480	55.25	9.16	Endoplasmic reticulum
**CYP92(3)**	CYP92A29	Medtr8g020940	7414776..7417453	474	54.68	9.17	Plasma membrane
	CYP92A90	Medtr8g020950	7422331..7426441	512	59.13	8.71	Plasma membrane
	CYP92A91	Medtr8g020960	7428665..7432354	514	59.45	8.97	Plasma membrane
**CYP93(18)**	CYP93A71	Medtr0171s0010	10159..12430	514	59.80	9.03	Plasma membrane
	CYP93A72	Medtr0171s0020	18352..20101	515	59.35	8.98	Plasma membrane
	CYP93A73	Medtr0171s0030	24025..26193	513	59.00	8.70	Plasma membrane
	CYP93A74	Medtr0181s0030	9535..12006	425	49.04	8.31	Plasma membrane
	CYP93A76	Medtr8g468780	24939523..24941318	506	57.75	8.69	Plasma membrane

	CYP93A77	Medtr8g469300	25167935..25169754	443	50.57	7.56	Plasma membrane
	CYP93A78	Medtr3g020780	6026235..6028627	512	58.60	7.59	Plasma membrane
	CYP93A8	Medtr7g092620	36733390..36735626	514	58.43	6.53	Plasma membrane
	CYP93B10	Medtr7g027960	9380852..9383322	511	58.28	6.96	Plasma membrane
	CYP93B11	Medtr7g028020	9398278..9400728	520	59.33	7.58	Plasma membrane
	CYP93B12	Medtr1g107295	48591920..48593768	520	59.17	7.17	Plasma membrane
	CYP93B28	Medtr4g062500	23222689..23225189	528	60.41	7.63	Plasma membrane
	CYP93B29	Medtr4g062510	23230337..23232426	526	59.89	7.94	Plasma membrane
	CYP93B30	Medtr4g062530	23243027..23244923	531	60.66	8.05	Plasma membrane
	CYP93C19	Medtr4g088170	34761467..34764335	440	50.24	8.95	Plasma membrane
	CYP93C20	Medtr4g088195	34779643..34782159	523	59.23	8.71	Plasma membrane
	CYP93C27	Medtr4g088160	34754100..34756270	522	59.02	8.57	Plasma membrane
	CYP93E2	Medtr7g056103	19505919..19508880	522	58.98	8.11	Plasma membrane
	CYP94A14	Medtr1g070345	31077053..31079090	514	58.16	8.87	Plasma membrane
	CYP94A51	Medtr7g096320	38641645..38643422	509	58.24	9.16	Endoplasmic reticulum
	CYP94A52	Medtr7g096340	38654452..38656449	505	58.32	9.13	Endoplasmic reticulum
	CYP94A53	Medtr1g070365	31126424..31128006	504	58.21	9.29	Endoplasmic reticulum
	CYP94A54	Medtr1g070370	31131654..31132382	509	57.80	7.67	Endoplasmic reticulum
**CYP94(11)**	CYP94B54	Medtr7g029275	10335207..10337443	511	59.23	8.93	Plasma membrane
	CYP94B55	Medtr3g080580	36463292..36465022	529	60.49	8.84	Plasma membrane
	CYP94C10	Medtr1g060550	26347876..26349522	515	59.34	8.50	Plasma membrane
	CYP94C60	Medtr2g086040	36169420..36170996	498	57.68	8.95	Plasma membrane
	CYP94C9	Medtr4g054920	19931469..19933167	517	58.90	8.64	Plasma membrane
	CYP94D24	Medtr7g088330	34391715..34393722	508	58.38	6.85	Plasma membrane
	CYP96A87	Medtr6g004590	336546..338099	517	59.40	7.26	Plasma membrane
	CYP96A88	Medtr6g004600	340584..342137	517	59.60	6.61	Plasma membrane
	CYP96A89	Medtr6g004610	346616..348046	476	55.50	8.52	Plasma membrane
	CYP96A90	Medtr6g004630	355308..356825	505	59.04	8.69	Plasma membrane
	CYP96A91	Medtr6g004670	376288..378006	505	58.66	8.11	Plasma membrane
	CYP96A92	Medtr7g033570	12163902..12165647	496	57.24	7.27	Plasma membrane
	CYP96A93	Medtr7g033580	12175607..12177097	496	57.31	8.42	Plasma membrane
	CYP96A94	Medtr8g088470	36667934..36669415	493	56.94	8.27	Plasma membrane
	CYP96A95	Medtr4g012320	3321253..3321930	225	25.53	8.25	Plasma membrane
**CYP96(24)**	CYP96J1	Medtr1g035940	13129637..13131166	509	59.17	7.89	Plasma membrane
	CYP96J10	Medtr1g035800	13085922..13087460	512	59.76	9.10	Plasma membrane
	CYP96J11	Medtr1g035830	13094208..13095737	509	59.29	6.99	Plasma membrane
	CYP96J12	Medtr1g035860	13100427..13102607	498	58.12	8.50	Plasma membrane
	CYP96J14	Medtr1g036200	13243399..13244904	501	58.10	8.69	Plasma membrane
	CYP96J15	Medtr1g036270	13271398..13272939	513	58.90	8.59	Plasma membrane
	CYP96J17	Medtr1g050362	19278022..19279548	508	58.77	7.92	Plasma membrane
	CYP96J18	Medtr1g069715	30367208..30373894	677	77.10	8.79	Extracellular (Secreted)
	CYP96J2	Medtr1g036010	13161330..13162859	509	59.21	8.38	Plasma membrane
	CYP96J4	Medtr1g036130	13204268..13205794	508	59.01	8.49	Plasma membrane

	CYP96J5	Medtr1g035580	12962762..12964258	498	57.65	8.61	Plasma membrane
	CYP96J6	Medtr1g035790	13079734..13081248	504	58.43	8.65	Plasma membrane
	CYP96J7	Medtr1g035670	13007009..13008523	504	58.26	8.49	Endoplasmic reticulum
	CYP96J8	Medtr1g035750	13064073..13065679	507	58.25	8.45	Extracellular (Secreted)
	CYP96J9	Medtr1g035760	13069045..13070559	481	55.98	7.97	Plasma membrane
	CYP97A10	Medtr7g079440	30155362..30162536	689	76.98	6.35	Membrane bound Chloroplast
**CYP97(3)**	CYP97B13	Medtr5g009110	2095507..2103878	574	64.44	6.35	Membrane bound Chloroplast
	CYP97C10	Medtr1g062190	27135478..27141580	541	60.60	5.71	Membrane bound Chloroplast
	CYP71D56P	Medtr5g094460.1					
	CYP71D57P	Medtr5g094500.1					
	CYP71D58P	Medtr5g094510.1					
	CYP71D60P	Medtr5g094535.1					
	CYP71D67P	Medtr5g094670.1					
	CYP71D68P	Medtr5g094790.1					
	CYP71D71Pa	Medtr3g057870.1					
	CYP71D71Pb	Medtr3g057880.1					
	CYP71D76P	Medtr3g057990.1					
	CYP71D80P	Medtr3g058150.1					
	CYP71D83P	Medtr5g073240.1					
	CYP71D84P	Medtr5g073230.1					
	CYP71D88P	Medtr5g095220.1					
	CYP71D91P	Medtr5g094840.1					
	CYP71D419P	Medtr8g016550.1					
	CYP71D438P	Medtr3g057170.1					
	CYP71AU60P	Medtr4g104570.1					
**Pseudogene (54)**	CYP72A63P	Medtr8g042040.1					
	CYP72A339P	Medtr2g072330.1					
	CYP75B84P	Medtr3g025250.1					
	CYP76E20P	Medtr7g075570.1					
	CYP76F75P	Medtr1g031710.1					
	CYP76F75P	Medtr0608s0010.1					
	CYP76X2P	Medtr1g090987.1					
	CYP76X11P	Medtr5g093950.1					
	CYP76X11P	Medtr5g093960.1					
	CYP76X17P	Medtr2g073480.1					
	CYP79D46P	Medtr8g075500.1					
	CYP82A10P	Medtr6g008630.1					
	CYP82C50P	Medtr5g018500.1					
	CYP82D995P	Medtr4g088405.1					
	CYP88A13P	Medtr5g014230.1					
	CYP84A53P	Medtr5g014850.1					
	CYP84A64P	Medtr4g471620.1					
	CYP93A75P	Medtr8g092760.1					

	CYP94C61P	Medtr2g061730.1
	CYP94C62P	Medtr2g061740.1
	CYP94C63P	Medtr2g061750.1
	CYP94C64P	Medtr2g061760.1
	CYP94C65P	Medtr2g062670.1
	CYP94C66P	Medtr2g062670.1b
	CYP94C67P	Medtr2g062680.1
	CYP96J3P	Medtr1g050590.1
	CYP96J13P	Medtr1g036140.1
	CYP96J16P	Medtr1g040110.1
	CYP704G4P	Medtr8g035790.1
	CYP704G8P	Medtr1g102210.1
	CYP704G17P	Medtr8g046580.1
	CYP721A39P	Medtr6g453220.1
	CYP728B30P	Medtr3g051230
	CYP736A128P	Medtr0451s0010.1
	CYP736A138P	Medtr6g084900.1
	CYP736A138P	Medtr6g084910.1
	CYP736A141P	Medtr8g104030.1
**Fragments (44)**	CYP71D-fragment1	Medtr8g015420.1
	CYP71D-fragment2	Medtr2g097840.1
	CYP71AU-fragment 1	Medtr1750s0010.1
	CYP72A-fragment_1	Medtr2g072420.1
	CYP72A-fragment_2	Medtr2g055530.1
	CYP72A-fragment_3	Medtr2g072410.1
	CYP72-fragment_1	Medtr3g079240.1
	CYP76E-fragment_1	Medtr7g075630.1
	CYP78A-fragment_1	Medtr7g089600.1
	CYP78A-fragment_2	Medtr3g072930.1
	CYP79D-fragment _1	Medtr3g467250.1
	CYP82A-fragment_1	Medtr5g094590.1
	CYP82D-fragment_1	Medtr4g088420.1
	CYP82J-fragment_1	Medtr6g022460.1
	CYP83G-fragment_1	Medtr5g072960.1
	CYP84A-fragment_1	Medtr4g057645.1
	CYP86B-fragment_1	Medtr1g023040.1
	CYP93B-fragment_1	Medtr1g107300.1
	CYP93B-fragment_2	Medtr1g107305.1
	CYP94C-fragment_1	Medtr2g061910.1
	CYP94C-fragment_2	Medtr2g089230.1
	CYP96A-fragment_2	Medtr6g004570.1
	CYP96J-fragment_1	Medtr6g084040.1
	CYP704B11-de6b	Medtr5g019165.1

	CYP704G9-de5b	Medtr1g102260.1
	CYP704G-fragment_1	Medtr1g102390.1
	CYP706A-fragment_1	Medtr4g102290.1
	CYP706A-fragment_2	Medtr0155s0010.1
	CYP707A-fragment_1	Medtr1g038803.1
	CYP711A10-de5b	Medtr1g015850.1
	CYP714E-fragment_1	Medtr3g062900.1
	CYP724A fragment	Medtr7g033040.1
	CYP729A-fragment_1	Medtr0457s0020.1
	CYP729A-fragment_2	Medtr0045s0100.1
	CYP736A-fragment_1	Medtr5g070685.1
	CYP736A-fragment_2	Medtr5g070840.1
	CYP736A-fragment_3	Medtr6g084760.1
	CYP736A137 part a	Medtr5g070980.1
	CYP736A137 part b	Medtr5g070973.1
	CYP76F73	Medtr0608s0030.1
	CYP76F74	Medtr0608s0020.1
	CYP82A15	Medtr0408s0010.1
	CYP83E43	Medtr4g025940.1
	CYP714H6	Medtr0727s0010.1

Table S2. Comparison of A-type P450 families among *Medicago*, soybean, *Arabidopsis*, rice, poplar, wheat and maize.

Family	Medicago	Soybean	Arabidopsis	Poplar	Rice	Wheat	Maize
**A-Type**							
**CYP71 Clan**							
CYP71	59	55	54	54	80	404	56
CYP73	2	3	1	4	4	14	3
CYP75	6	7	1	3	3	26	5
CYP76	25	14	9	14	30	59	6
CYP77	2	4	6	4	2	5	2
CYP78	4	11	6	13	8	11	8
CYP79	8	5	13	6	4	24	4
**CYP80**	**0**	**0**	**0**	**6**	**0**	**0**	**0**
CYP81	9	12	16	42	10	43	12
**CYP82**	**19**	**24**	**5**	**30**	**0**	**0**	**0**
**CYP83**	**18**	**12**	**2**	**7**	**0**	**0**	**0**
CYP84	3	3	1	4	3	27	2
CYP89	12	8	7	19	19	55	16
**CYP92**	**3**	**2**	**0**	**13**	**9**	**19**	**11**
CYP93	18	13	1	9	4	15	7
CYP98	0	2	3	6	2	10	2
**CYP99**	**0**	**0**	**0**	**0**	**2**	**27**	**4**
CYP701	1	2	1	1	6	10	2
CYP703	1	1	1	2	1	2	1
**CYP705**	**0**	**0**	**31**	**0**	**0**	**0**	**0**
CYP706	2	3	7	5	2	3	2
CYP712	2	2	2	15	0	0	0
**CYP723**	**0**	**0**	**0**	**0**	**2**	**27**	**0**
CYP736	15	12	0	18	0	0	0
Total	209	195	167	275	191	781	143

Table S3. Comparison of non-A type P450 families among *Medicago*, soybean, *Arabidopsis*, poplar, rice, wheat and maize.

Family	Medicago	Soybean	Arabidopsis	Poplar	Rice	Wheat	Maize
**Non-A Type**							
**CYP51 clan**							
CYP51	1	2	2	2	10	37	7
**CYP72 clan**							
CYP72	15	12	10	16	14	58	16
CYP709	1	0	3	2	11	44	13
CYP714	6	6	2	7	6	22	5
CYP715	5	6	1	3	1	3	1
CYP721	2	2	1	11	2	7	2
**CYP749**	**0**	**0**	**0**	**16**	**0**	**0**	**0**
CYP734	1	3	2	2	4	12	5
CYP735	2	3	4	2	2	6	3
**CYP74 clan**							
CYP74	5	6	2	9	5	15	5
**CYP85 clan**							
CYP85	3	5	1	4	1	14	1
CYP87	1	2	1	17	10	29	8
CYP88	10	3	2	3	1	3	3
CYP90	7	12	4	9	6	12	5
**CYP702**	**0**	**0**	**8**	**0**	**0**	**0**	**0**
CYP707	6	10	4	8	5	6	5
**CYP708**	**0**	**0**	**3**	**0**	**0**	**0**	**0**
**CYP716**	**2**	**7**	**2**	**25**	**0**	**0**	**0**
**CYP718**	**1**	**1**	**1**	**2**	**0**	**0**	**0**
**CYP720**	**1**	**2**	**1**	**1**	**0**	**0**	**0**
CYP722	2	2	1	2	1	3	1
CYP724	0	1	1	2	1	3	1
**CYP728**	**3**	**2**	**0**	**14**	**11**	**16**	**4**
CYP729	3	0	0	1	3	9	0
**CYP733**	**1**	**3**	**0**	**1**	**1**	**2**	**1**
**CYP86 clan**							
CYP86	8	9	10	9	7	16	7
CYP94	11	14	6	16	22	46	12
CYP96	24	7	14	11	11	82	7
CYP704	9	5	3	9	7	28	5
**CYP97 clan**							
CYP97	3	5	3	3	11	12	3
**CYP710 clan**							
CYP710	1	2	4	1	4	7	1
**CYP711 clan**							

CYP711	2	4	1	3	6	12	4
**CYP727 clan**							
CYP727	1	1	0	2	1	3	2
Total	137	137	97	213	164	507	127

Table S4 All tandem and segmental duplicated *MtP450* gene pairs in *M. truncatula.*

Duplicated Type	Gene Name	Gene Name	Ka	Ks	Ka/Ks	Effective Len	Average S-sites	Average N-sites	Selection pressure
Tandem	*MtCYP93A71*	*MtCYP93A72*	0.129	0.430	0.301	1536	328.33	1207.67	Purifying selection
Tandem	*MtCYP93A72*	*MtCYP93A73*	0.138	0.418	0.331	1275	267.83	1007.17	Purifying selection
Tandem	*MtCYP83D4*	*MtCYP83D3*	0.034	0.132	0.257	1542	338.42	1203.58	Purifying selection
Tandem	*MtCYP96J8*	*MtCYP96J9*	0.207	0.534	0.387	1440	308.00	1132.00	Purifying selection
Tandem	*MtCYP96J6*	*MtCYP96J10*	0.128	0.453	0.283	1506	316.92	1189.08	Purifying selection
Tandem	*MtCYP86B18*	*MtCYP86B19*	0.074	0.263	0.280	1665	350.75	1314.25	Purifying selection
Tandem	*MtCYP94A53*	*MtCYP94A54*	0.091	0.521	0.174	726	161.50	564.50	Purifying selection
Tandem	*MtCYP76X13*	*MtCYP76X14*	0.061	0.215	0.283	1452	318.75	1133.25	Purifying selection
Tandem	*MtCYP715A26*	*MtCYP715A27*	0.072	0.193	0.373	1536	328.92	1207.08	Purifying selection
Tandem	*MtCYP76F76*	*MtCYP76F77*	0.100	0.663	0.150	1497	335.08	1161.92	Purifying selection
Tandem	*MtCYP89A29*	*MtCYP89A30*	0.076	0.171	0.445	1464	319.83	1144.17	Purifying selection
Tandem	*MtCYP89A30*	*MtCYP89A31*	0.058	0.132	0.442	1422	309.67	1112.33	Purifying selection
Tandem	*MtCYP89A31*	*MtCYP89A32*	0.092	0.300	0.306	1470	323.67	1146.33	Purifying selection
Tandem	*MtCYP89A32*	*MtCYP89A33*	0.066	0.322	0.204	1512	335.92	1176.08	Purifying selection
Tandem	*MtCYP88A55*	*MtCYP88A54*	0.138	0.812	0.170	1461	322.00	1139.00	Purifying selection
Tandem	*MtCYP72A336*	*MtCYP72A66*	0.060	0.291	0.207	1539	327.00	1212.00	Purifying selection
Tandem	*MtCYP72A59v1*	*MtCYP72A341*	0.082	0.322	0.255	1479	325.33	1153.67	Purifying selection
Tandem	*MtCYP76X16*	*MtCYP76X17*	0.069	0.245	0.282	1407	308.50	1098.50	Purifying selection
Tandem	*MtCYP71D74*	*MtCYP71D75*	0.093	0.354	0.262	1476	319.67	1156.33	Purifying selection
Tandem	*MtCYP71D422*	*MtCYP71D423*	0.136	0.473	0.287	1521	339.00	1182.00	Purifying selection
Tandem	*MtCYP71D424*	*MtCYP71D425*	0.100	0.324	0.309	1287	284.83	1002.17	Purifying selection
Tandem	*MtCYP71D425*	*MtCYP71D426*	0.056	0.239	0.234	1410	313.00	1097.00	Purifying selection
Tandem	*MtCYP71D428*	*MtCYP71D429*	0.113	0.336	0.336	1518	336.75	1181.25	Purifying selection

Tandem	*MtCYP71A60*	*MtCYP71A30*	0.347	1.051	0.330	1353	292.75	1060.25	Purifying selection
Tandem	*MtCYP728H6*	*MtCYP728H7*	0.104	0.303	0.345	1443	313.17	1129.83	Purifying selection
Tandem	*MtCYP83E39*	*MtCYP83E40*	0.032	0.016	2.020	933	202.50	730.50	Positive selection
Tandem	*MtCYP83E40*	*MtCYP83E8*	0.100	0.315	0.319	1488	331.75	1156.25	Purifying selection
Tandem	*MtCYP83E41*	*MtCYP83E42*	0.105	0.545	0.192	1035	223.17	811.83	Purifying selection
Tandem	*MtCYP93B28*	*MtCYP93B29*	0.121	0.593	0.204	1578	351.58	1226.42	Purifying selection
Tandem	*MtCYP93C27*	*MtCYP93C19*	0.041	0.300	0.136	1563	362.17	1200.83	Purifying selection
Tandem	*MtCYP71AU56*	*MtCYP71AU57*	0.130	0.343	0.379	1488	334.00	1154.00	Purifying selection
Tandem	*MtCYP71AU57*	*MtCYP71AU58*	0.106	0.290	0.364	1554	351.17	1202.83	Purifying selection
Tandem	*MtCYP81E59*	*MtCYP81E7*	0.303	1.341	0.226	1461	329.83	1131.17	Purifying selection
Tandem	*MtCYP71AU60*	*MtCYP71AU61*	0.134	0.322	0.416	1290	286.17	1003.83	Purifying selection
Tandem	*MtCYP71A31*	*MtCYP71A61*	0.151	0.324	0.468	1545	335.42	1209.58	Purifying selection
Tandem	*MtCYP71D431*	*MtCYP71D432*	0.106	0.270	0.392	1155	255.42	899.58	Purifying selection
Tandem	*MtCYP76X3*	*MtCYP76X4*	0.045	0.226	0.198	1401	309.58	1091.42	Purifying selection
Tandem	*MtCYP88A13*	*MtCYP88A14*	0.122	0.300	0.406	1467	326.25	1140.75	Purifying selection
Tandem	*MtCYP81E8v2*	*MtCYP81E10*	0.287	1.380	0.208	1473	323.33	1149.67	Purifying selection
Tandem	*MtCYP71D85*	*MtCYP71D86*	0.203	0.622	0.326	1512	332.67	1179.33	Purifying selection
Tandem	*MtCYP71D86*	*MtCYP71D87*	0.149	0.450	0.333	1515	329.00	1186.00	Purifying selection
Tandem	*MtCYP736A134*	*MtCYP736A135*	0.079	0.197	0.399	1524	337.67	1186.33	Purifying selection
Tandem	*MtCYP736A135*	*MtCYP736A132b*	0.097	0.305	0.319	1527	337.00	1190.00	Purifying selection
Tandem	*MtCYP736A133*	*MtCYP736A134*	0.089	0.239	0.372	1272	286.08	985.92	Purifying selection
Tandem	*MtCYP736A132b*	*MtCYP736A136*	0.107	0.333	0.320	1524	339.00	1185.00	Purifying selection
Tandem	*MtCYP71D61*	*MtCYP71D62*	0.090	0.263	0.343	1506	328.25	1177.75	Purifying selection
Tandem	*MtCYP71D62*	*MtCYP71D63*	0.053	0.156	0.340	1506	324.83	1181.17	Purifying selection
Tandem	*MtCYP71D63*	*MtCYP71D64*	0.125	0.334	0.374	1506	327.42	1178.58	Purifying selection
Tandem	*MtCYP96A87*	*MtCYP96A88*	0.058	0.215	0.271	1551	329.00	1222.00	Purifying selection

Tandem	*MtCYP96A88*	*MtCYP96A89*	0.217	0.775	0.280	1392	288.58	1103.42	Purifying selection
Tandem	*MtCYP96A89*	*MtCYP96A90*	0.055	0.141	0.387	1395	285.50	1109.50	Purifying selection
Tandem	*MtCYP82A8*	*MtCYP82A9*	0.124	0.321	0.388	1590	359.17	1230.83	Purifying selection
Tandem	*MtCYP82A11*	*MtCYP82A12*	0.088	0.157	0.562	1587	354.92	1232.08	Purifying selection
Tandem	*MtCYP96A92*	*MtCYP96A93*	0.066	0.202	0.329	1488	312.67	1175.33	Purifying selection
Tandem	*MtCYP76E1*	*MtCYP76E22*	0.103	0.305	0.339	1506	334.58	1171.42	Purifying selection
Tandem	*MtCYP82L14*	*MtCYP82J8*	0.463	1.343	0.345	1314	290.50	1023.50	Purifying selection
Tandem	*MtCYP92A29*	*MtCYP92A90*	0.080	0.399	0.201	1533	343.33	1189.67	Purifying selection
Tandem	*MtCYP92A90*	*MtCYP92A91*	0.076	0.320	0.237	1539	338.83	1200.17	Purifying selection
Tandem	*MtCYP704G2*	*MtCYP704G16*	0.037	0.165	0.226	1530	328.33	1201.67	Purifying selection
Tandem	*MtCYP79D13*	*MtCYP79D12*	0.022	0.071	0.313	1581	352.58	1228.42	Purifying selection
Tandem	*MtCYP736A139*	*MtCYP736A140*	0.117	0.320	0.366	1308	285.58	1022.42	Purifying selection
Segmental	*MtCYP74A1*	*MtCYP74A37*	0.244	1.787	0.136	1569	362.83	1206.17	Purifying selection
Segmental	*MtCYP94A14*	*MtCYP94A51*	0.326	1.614	0.202	1506	334.92	1171.08	Purifying selection
Segmental	*MtCYP71AP16*	*MtCYP71AU62*	0.467	2.563	0.182	1461	332.00	1129.00	Purifying selection
Segmental	*MtCYP71A60*	*MtCYP71A31*	0.343	1.124	0.305	1350	291.75	1058.25	Purifying selection
Segmental	*MtCYP90A14*	*MtCYP90A40*	0.098	0.811	0.121	1413	326.67	1086.33	Purifying selection
Segmental	*MtCYP81E58*	*MtCYP81E8v2*	0.229	1.783	0.128	1494	334.00	1160.00	Purifying selection
Segmental	*MtCYP90B11*	*MtCYP90B10*	0.122	0.696	0.175	1434	306.83	1127.17	Purifying selection
Segmental	*MtCYP90C20*	*MtCYP90C4*	0.159	0.696	0.229	1431	308.42	1122.58	Purifying selection
Segmental	*MtCYP84A17*	*MtCYP84A18*	0.142	1.439	0.099	1521	343.58	1177.42	Purifying selection
Segmental	*MtCYP707A17*	*MtCYP707A19*	0.098	0.634	0.154	1392	308.08	1083.92	Purifying selection

Table S5 One-to-one orthologous relationships of *P450* genes between *Medicago* and *Arabidopsis*.

***MtP450* genes**	***AtP450* genes**	**Ka**	**Ks**	**Ka/Ks**	**Effective Len**	**Average S-sites**	**Average N-sites**	**Selection pressure**
MtCYP721A40	*AtCYP721A1*	0.445	2.030	0.219	1500	333.83	1166.17	Purifying selection
MtCYP97C10	*AtCYP97C1*	0.171	1.699	0.101	1614	382.75	1231.25	Purifying selection
MtCYP709B4	*AtCYP709B2*	0.366	NaN	NaN	1542	337.83	1204.17	/
MtCYP715A26	*AtCYP715A1*	0.290	NaN	NaN	1524	330.92	1193.08	/
MtCYP94C60	*AtCYP94C1*	0.311	3.248	0.096	1458	327.42	1130.58	Purifying selection
MtCYP94C60	*OsCYP94C2*	0.431	NaN	NaN	1494	352.33	1141.67	/
MtCYP90A14	*AtCYP90A1*	0.161	2.254	0.071	1410	328.83	1081.17	Purifying selection
MtCYP71AP16	*AtCYP71A25*	0.554	NaN	NaN	1467	333.50	1133.50	/
MtCYP71A60	*AtCYP71A26*	0.567	3.444	0.165	1254	279.50	974.50	Purifying selection
MtCYP722A1	*AtCYP722A1*	0.351	2.096	0.168	1419	311.50	1107.50	Purifying selection
MtCYP86A94	*AtCYP86A1*	0.209	3.096	0.068	1500	358.67	1141.33	Purifying selection
MtCYP707A106	*AtCYP707A4*	0.229	NaN	NaN	1401	313.33	1087.67	/
MtCYP81E58	*AtCYP81D7*	0.411	2.716	0.151	1488	339.75	1148.25	Purifying selection
MtCYP81E58	*AtCYP81H1*	0.437	1.980	0.221	1506	345.92	1160.08	Purifying selection
MtCYP81E58	*AtCYP81G1*	0.468	2.560	0.183	1473	344.33	1128.67	Purifying selection
MtCYP75B86	*AtCYP75B1*	0.273	NaN	NaN	1515	359.75	1155.25	/
MtCYP77B5	*AtCYP77B1*	0.236	2.457	0.096	1518	347.58	1170.42	Purifying selection
MtCYP87A9	*AtCYP87A2*	0.185	4.783	0.039	1416	310.42	1105.58	Purifying selection
MtCYP81E8v2	*AtCYP81D7*	0.393	NaN	NaN	1485	336.42	1148.58	/
MtCYP81E8v2	*AtCYP81H1*	0.436	2.763	0.158	1488	339.42	1148.58	Purifying selection
MtCYP81E8v2	*AtCYP81G1*	0.464	NaN	NaN	1467	338.75	1128.25	/
MtCYP84A17	*AtCYP84A1*	0.176	NaN	NaN	1542	352.92	1189.08	/
MtCYP73A3	*AtCYP73A5*	0.117	3.109	0.038	1515	338.58	1176.42	Purifying selection
MtCYP90A40	*AtCYP90A1*	0.151	1.978	0.076	1404	330.67	1073.33	Purifying selection

MtCYP712B1	*AtCYP712A1*	0.438	NaN	NaN	1533	343.00	1190.00	/
MtCYP712B1	*AtCYP712A2*	0.480	NaN	NaN	1512	342.92	1169.08	/
MtCYP93A8	*AtCYP93D1*	0.386	2.000	0.193	1491	328.83	1162.17	Purifying selection
MtCYP76A39	*AtCYP76G1*	0.546	3.496	0.156	1524	346.50	1177.50	Purifying selection
MtCYP78A29	*AtCYP78A8*	0.285	NaN	NaN	1578	368.25	1209.75	/
MtCYP78A29	*AtCYP78A6*	0.261	2.171	0.120	1569	367.67	1201.33	Purifying selection
MtCYP78A29	*AtCYP78A9*	0.246	2.574	0.096	1569	369.50	1199.50	Purifying selection
MtCYP86A24	*AtCYP86A8*	0.161	NaN	NaN	1602	375.50	1226.50	/
MtCYP86A24	*AtCYP86A2*	0.187	2.999	0.062	1611	375.67	1235.33	Purifying selection
MtCYP704G6	*AtCYP704A2*	0.394	NaN	NaN	1506	327.08	1178.92	/
MtCYP84A18	*AtCYP84A1*	0.205	NaN	NaN	1527	350.50	1176.50	/
MtCYP86A93	*AtCYP86A7*	0.212	2.114	0.100	1551	356.75	1194.25	Purifying selection

Table S6 One-to-one orthologous relationships of *P450* genes between *Medicago* and rice.

***MtP450* Gene**	***OsP450* Gene**	**Ka**	**Ks**	**Ka/Ks**	**Effective Len**	**Average S-sites**	**Average N-sites**	**Selection pressure**
MtCYP94C60	*OsCYP94C2*	0.431	NaN	NaN	1494	352.33	1141.67	/

Table S7.Motif with best possible match and its width.

Name	Best possible match	Width
motif-1	KGQNFEFIPFGAGRRICPGINLA	23
motif-2	KLPYLKAVIKETLRLHP	17
motif-3	AGTDTTSSTJEWAMA	15
motif-4	ASEDCEINGYHIPKGTRVLVNAWAIGR	27
motif-5	SKKYGPJMHLKLGSVPTIVVSSPEMA	26
motif-6	VLKTHDLVFASRPKLLASK	19
motif-7	WEDPEEFKPER	11
motif-8	FAPYGPYWRQLRKICTLELLS	21
motif-9	ELLKNPRVMKKAQEEIRE	18
motif-10	NVELVLANLLYHFDWKLPNGMKPEDLD	27

Table S8. Name of the primers used in the present study for qPCR.

Primer name	Sequence(5' to 3')
CYP83D3qRTF	TGGGGTTTCTTGTGCTTCTTTCT
CYP83D3qRTR	TGTGGGGATGAAGGGTCAAG
CYP76F70qRTF	GGACGCACTCTCCGTTCTC
CYP76F70qRTR	TAGCGCCTGGCTTGAATCG
CYP72A66qRTF	ATGGTCTTGGAAGGTGCTGAA
CYP72A66qRTR	ACTCCTTCATGTCTCCAACTAAA
CYP76E1qRTF	CCTTCGCCAACAAAAGTTGC
CYP76E1qRTR	TCAGGTACTGTGGAATGTGCC
CYP74C12qRTF	TGCGAATTGAACCTGCTGTG
CYP74C12qRTR	AACTTCTCACCTTCGCCGAC
CYP94A52qRTF	GTGGTTGACACGGAACTCAC
CYP94A52qRTR	CGTTCTTTTAGCCGACGGAGT
CYP72A59v1qRTF	CCAACACCAAGGGTGAACAT
CYP72A59v1qRTR	TTGGTAACATGATAAGACCATTAGC
CYP74B4V1qRTF	ATCTTCCTCCATTCCTTCTCTTACC
CYP74B4V1qRTR	CATCTTCACTCAACCCAAACTCTG
CYP71AU56qRTF	TTCCACTCTTACTCTCTCTTCTGT
CYP71AU56qRTR	TGGAGGTGAAGGTGGTAAGTG
CYP81E9qRTF	CATTGAGGAACATCGCCGTG
CYP81E9qRTR	CCGCAAGAAGCATACCCTGA
CYP71A31qRTF	ATGAAGGAGATAATGAAGGCAACG
CYP71A31qRTR	TCCACTAAGAACATCAATCCAACC
CYP704G6qRTF	GGCCGCTACACTTTCTTGGT
CYP704G6qRTR	TGAGAGTCTCTGTGAGTGTTGC
CYP76Y14qRTF	TGAATGGGCAATGGCAGAGC
CYP76Y14qRTR	GGATGTATGGAAGTGAAGGAATGTC
CYP78A126qRTF	TGCAGGTCGTAGGGTTTGTC
CYP78A126qRTR	AGGTACTTGTGCTGGAAGCC

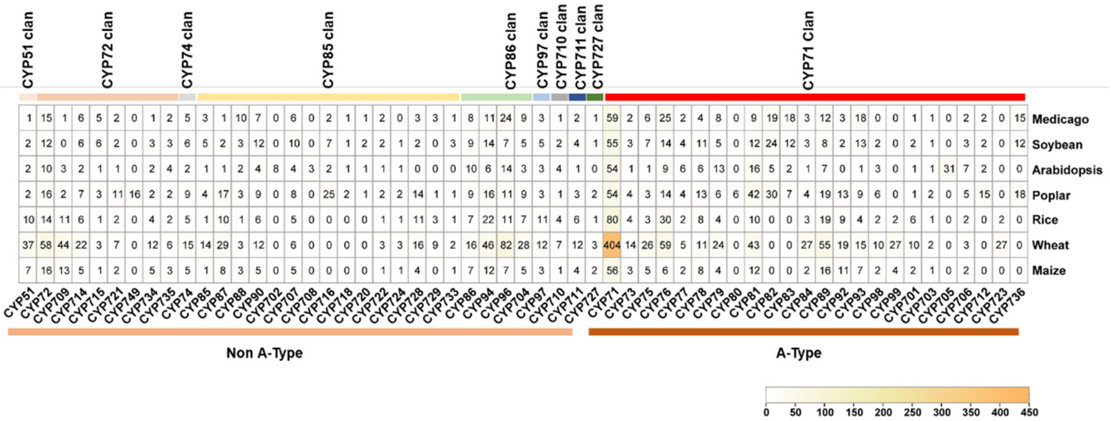

Figure S1. Comparison of P450 family numbers among *Medicago*, soybean, *Arabidopsis*, poplar, rice, wheat and maize. P450 clans and types were labeled with lines in different colors.

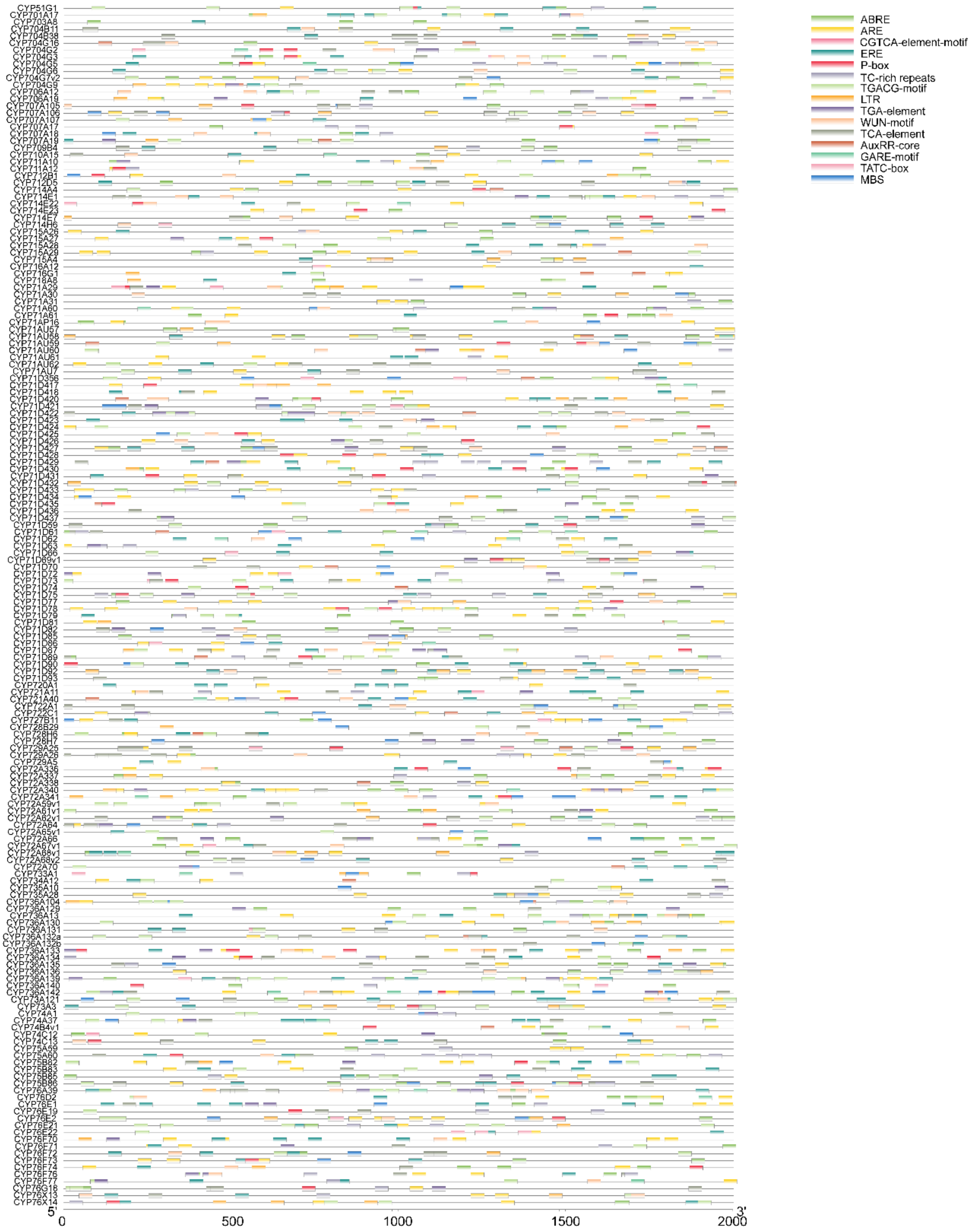
ABRE
ARE
CGTCA-element-motif
ERE
P-box
TC-rich repeats
TGACG-motif
LTR
TGA-element
WUN-motif
TCA-element
AuxRR-core
GARE-motif
TATC-box
MBS
5'
3'
0
500
1000
1500
2000

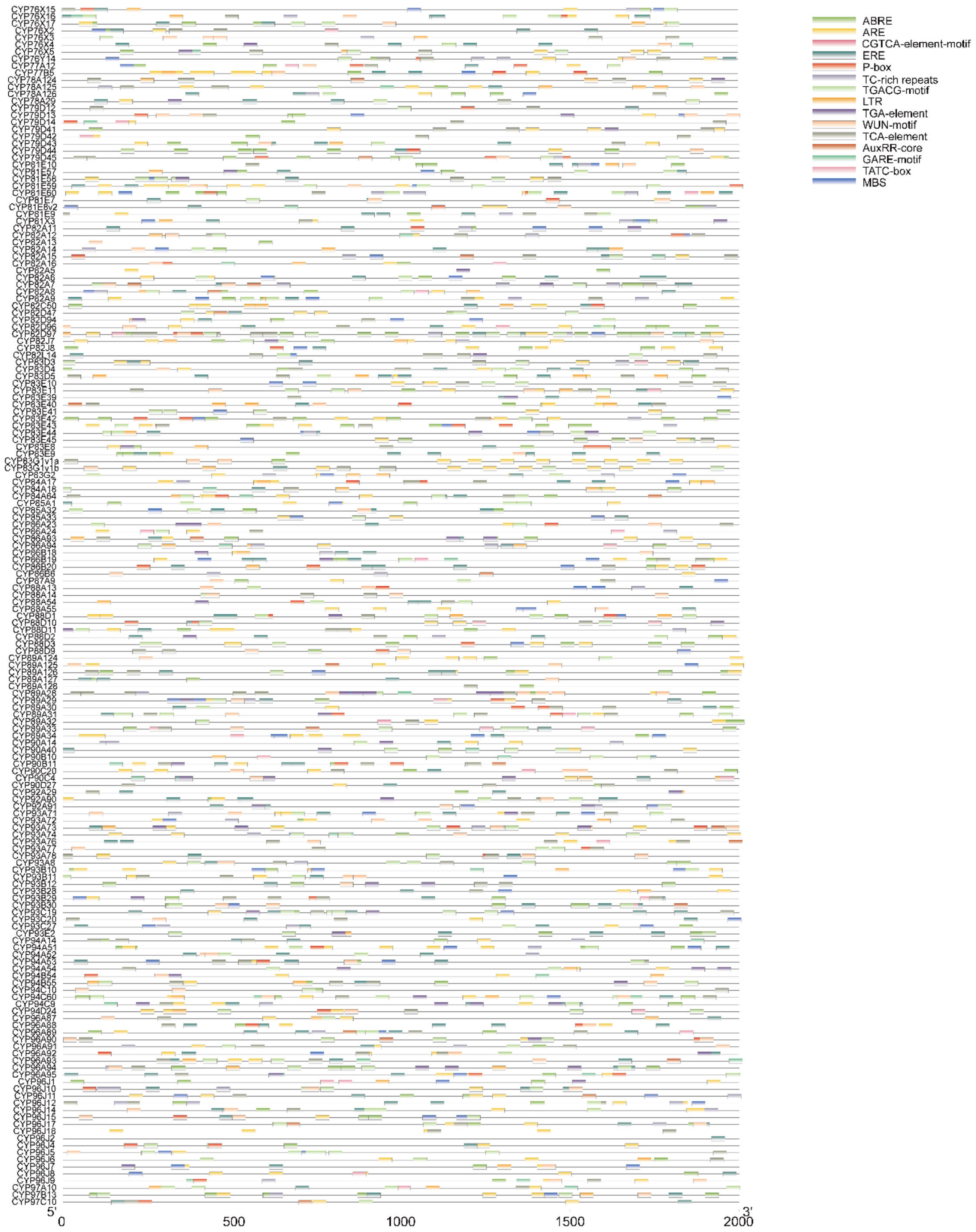

Figure S2. Analysis of the *cis*-acting elements of *MtP450*s. The different colored block represented different types of *cis*-acting elements and their locations in each *MtP450* gene.

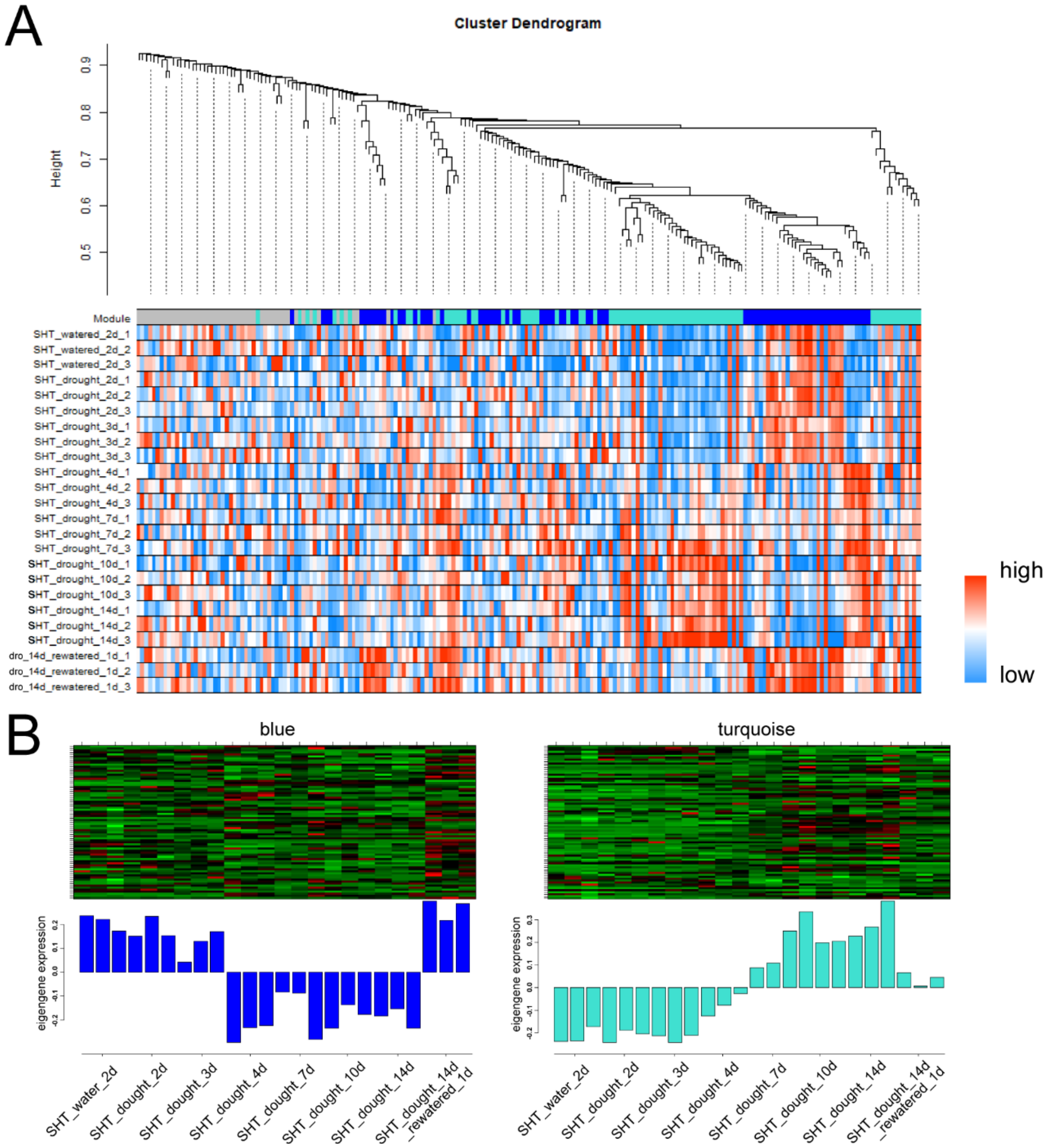

Figure S3. Construction of co-expression network of *MtP450* genes under drought treatment in shoots. A: Hierarchical clustering dendrogram of topological overlaps of *MtP450* genes of shoot under drought treatments from microarray data. Red represents the level of gene expression is higher and blue represents the level of gene expression is lower in each sample about the color bar. B: Expression pattern of the genes and eigengenes of each module. The heatmap was plotted using the log10 FPKM values.

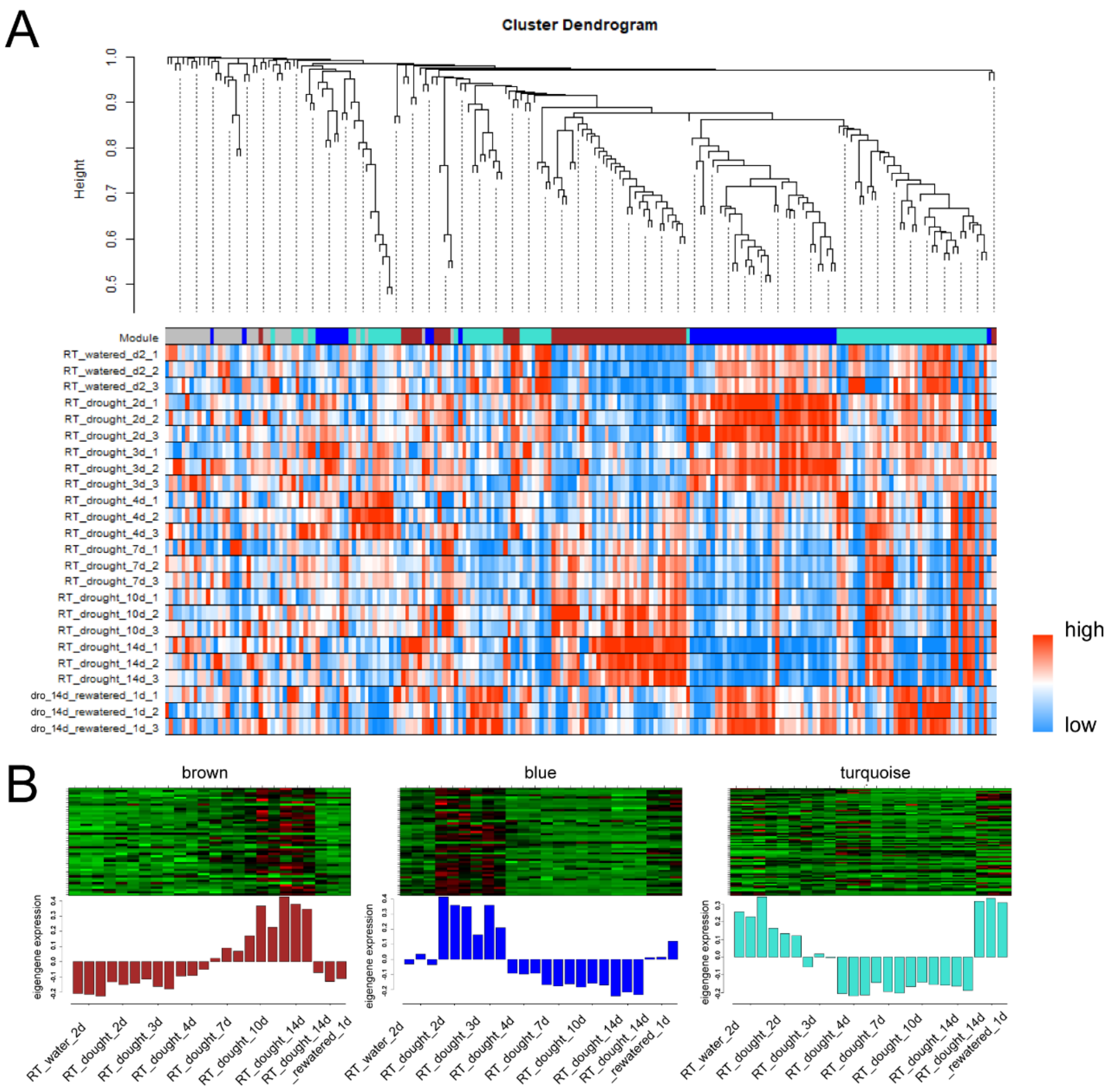

Figure S4. Construction of co-expression network of *MtP450* genes under drought treatment in roots. A: Hierarchical clustering dendrogram of topological overlaps of *MtP450*s genes of root under drought treatments from microarray data. Red represents the level of gene expression is higher and blue represents the level of gene expression is lower in each sample about the color bar. B: Expression pattern of the genes and eigengenes of each module. The heatmap was plotted using the log10 FPKM values.

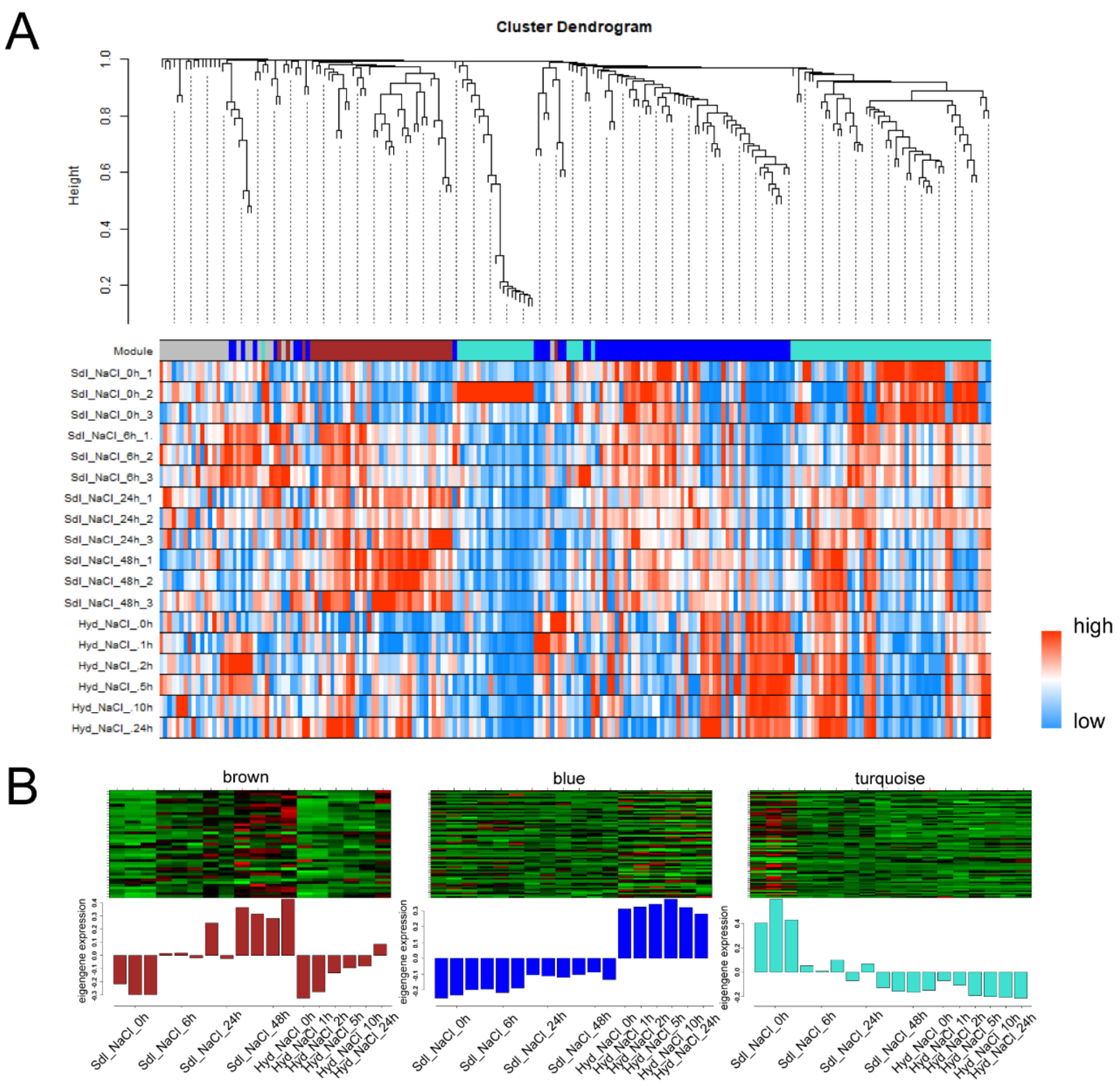

Figure S5. Construction of co-expression network of *MtP450* genes under NaCl treatments. A: Hierarchical clustering dendrogram of topological overlaps of *MtP450*s genes under NaCl treatments from microarray data. The cut-tree hybrid method was used to pick a height cut-off and to identify modules, which are shown in the panel below the dendrogram. Red represents the level of gene expression is higher and blue represents the level of gene expression is lower in each sample about the color bar. Each module is labeled with a unique color for easy visualization and understanding. B: Expression pattern of the genes and eigengenes of each module. The heatmap was plotted using the log10 FPKM values.

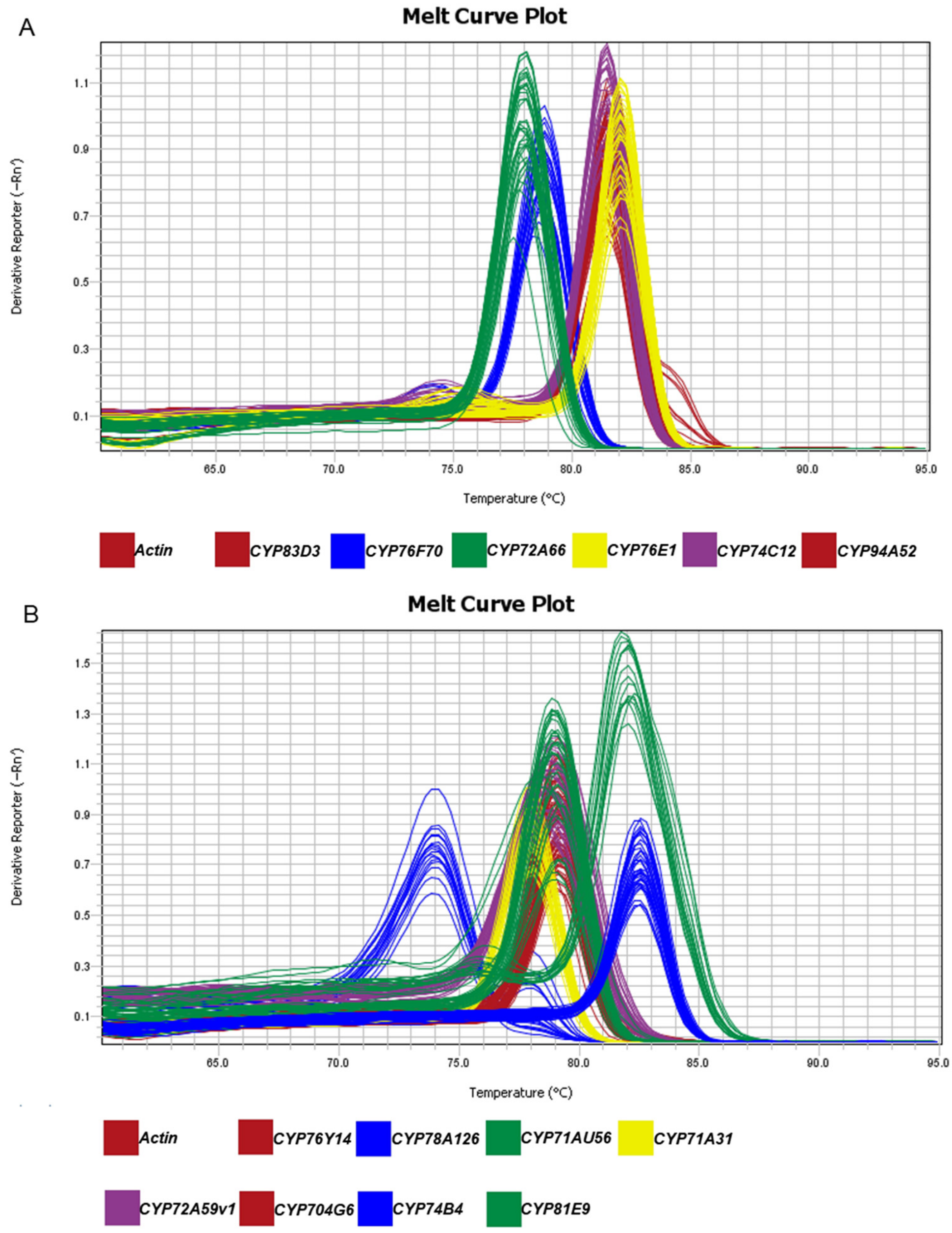

Figure S6. Melting curve analysis of the *MtP450* hub genes using samples under NaCl and PEG treatments. A: The melting curve analysis of the hub genes (*CYP83D3*, *CYP76F70*, *CYP72A66*, *CYP76E1*, *CYP74C12*, *CYP94A52*) and *actin* gene using samples under NaCl treatments. B: The melting curve analysis of the hub genes (*CYP76Y14*, *CYP78A126*, *CYP71AU56*, *CYP71A31*, *CYP72A59V1*, *CYP704G6*, *CYP74B4*, *CYP81E9*) and *actin* gene using samples under PEG treatments.
